# The collagen ColQ binds to LRP4 and regulates the activation of the Muscle-Specific Kinase–LRP4 receptor complex by agrin at the neuromuscular junction

**DOI:** 10.1016/j.jbc.2023.104962

**Published:** 2023-06-23

**Authors:** Thi Minh Uyen Dao, Susie Barbeau, Julien Messéant, Bruno Della-Gaspera, Tahar Bouceba, Fannie Semprez, Claire Legay, Alexandre Dobbertin

**Affiliations:** 1 Université Paris Cité, CNRS, Saints-Pères Paris Institute for the Neurosciences, Paris, France; 2 Université Paris Cité, INSERM UMR-S 1124, Paris, France; 3 Sorbonne Université, CNRS, IBPS, Protein Engineering Platform, Paris, France

**Keywords:** neuromuscular junction, myasthenic syndromes, ColQ, AChE, MuSK, LRP4, agrin

## Abstract

Collagen Q (ColQ) is a nonfibrillar collagen that plays a crucial role at the vertebrate neuromuscular junction (NMJ) by anchoring acetylcholinesterase to the synapse. ColQ also functions in signaling, as it regulates acetylcholine receptor clustering and synaptic gene expression, in a manner dependent on muscle-specific kinase (MuSK), a key protein in NMJ formation and maintenance. MuSK forms a complex with low-density lipoprotein receptor–related protein 4 (LRP4), its coreceptor for the proteoglycan agrin at the NMJ. Previous studies suggested that ColQ also interacts with MuSK. However, the molecular mechanisms underlying ColQ functions and ColQ–MuSK interaction have not been fully elucidated. Here, we investigated whether ColQ binds directly to MuSK and/or LRP4 and whether it modulates agrin-mediated MuSK–LRP4 activation. Using coimmunoprecipitation, pull-down, plate-binding assays, and surface plasmon resonance, we show that ColQ binds directly to LRP4 but not to MuSK and that ColQ interacts indirectly with MuSK through LRP4. In addition, we show that the LRP4 N-terminal region, which contains the agrin-binding sites, is also crucial for ColQ binding to LRP4. Moreover, ColQ–LRP4 interaction was reduced in the presence of agrin, suggesting that agrin and ColQ compete for binding to LRP4. Strikingly, we reveal ColQ has two opposing effects on agrin-induced MuSK–LRP4 signaling: it constitutively reduces MuSK phosphorylation levels in agrin-stimulated myotubes but concomitantly increases MuSK accumulation at the muscle cell surface. Our results identify LRP4 as a major receptor of ColQ and provide new insights into mechanisms of ColQ signaling and acetylcholinesterase anchoring at the NMJ.

The vertebrate neuromuscular junction (NMJ) is a cholinergic synapse, where the duration of the synaptic transmission is largely controlled by the enzyme acetylcholinesterase (AChE), which hydrolyzes the neurotransmitter acetylcholine. At the NMJ, most of the AChE is found associated with a specific collagen, called collagen Q (ColQ). These AChE–ColQ hetero-oligomers, also called asymmetric forms, play a critical role in accumulating and anchoring AChE in the synaptic basal lamina of the NMJ. ColQ is a nonfibrillar homotrimeric collagen composed of a central triple-helical collagenous domain flanked by noncollagenous N- and C-terminal domains (1, 2, 3, 4, 5). Each N-terminal domain contains a short proline-rich attachment domain, which is responsible for binding and organizing AChE tetramers. The collagenous domain, through its two heparin-binding domains, interacts critically with the heparan sulfate chains of the proteoglycan, perlecan, present in the synaptic basal lamina. Consequently, AChE–ColQ clusters are absent at the NMJs of perlecan null mice (6, 7, 8). The C-terminal domains of ColQ are each divided into a region important for triple-helix formation and a cysteine-rich region that plays a crucial role in AChE–ColQ accumulation at the NMJ. Thus, the two heparin-binding domains as well as the C-terminal domain of ColQ are required for the anchoring and clustering of AChE–ColQ at the NMJ (9, 10).

Patients carrying ColQ mutations and mice deficient for ColQ present with congenital myasthenic syndromes, a class of pathologies characterized by fatigable muscle weakness, with AChE deficiency (5, 11, 12, 13, 14, 15). Over 50 mutations in *COLQ* have been identified. Mutations in the collagenous or in the trimerization domain impair the formation of the triple helix, whereas those located in the proline-rich attachment domain prevent the association of AChE tetramers with ColQ. Numerous mutations have also been identified within the ColQ C terminus that do not abrogate the formation of the ColQ triple helix and AChE–ColQ hetero-oligomers but hinder the accumulation of AChE at the NMJ, suggesting that the ColQ C-terminal domain interacts with partners that anchor ColQ in the synapse. One of these partners is the tyrosine kinase receptor MuSK (muscle-specific kinase) (9, 16, 17, 18). In addition to its structural AChE anchoring role, ColQ exerts important regulatory functions at the synapse by controlling acetylcholine receptor (AChR) clustering and synaptic gene expression, through a mechanism that involves, at least in part, MuSK (19).

MuSK and its coreceptor LRP4 (low-density lipoprotein receptor–related protein 4), a single-transmembrane member of the low-density lipoprotein receptor family, play a crucial role at the NMJ (20, 21, 22, 23, 24). MuSK^−/−^ and LRP4^−/−^ mutant mice do not form NMJs and die shortly after birth (25, 26). Mutations in LRP4 or MuSK and autoantibodies directed against these proteins cause congenital myasthenic syndromes and myasthenia gravis, respectively (15, 27, 28, 29, 30, 31). The MuSK–LRP4 complex is required, before innervation, for muscle prepatterning, where it is stimulated in part by specific Wnt proteins and controls the formation of AChR microaggregates in a broad region in the middle of the muscle fiber that will become its synaptic domain (24, 32, 33, 34). Upon innervation, MuSK–LRP4 is further activated by neural agrin, a heparan sulfate proteoglycan secreted by motor nerve terminals, which instructs and stabilizes postjunctional differentiation and induces synapse-specific gene expression. Agrin binds to LRP4 but not to MuSK. This in turn increases the binding of LRP4 to MuSK, stimulating MuSK dimerization and autophosphorylation and subsequently initiating downstream signaling pathways necessary for postsynaptic differentiation and AChR clustering (24, 25, 35, 36, 37, 38). The agrin–LRP4–MuSK pathway is finely controlled, and MuSK activation leads to its internalization by endocytosis, which is necessary for AChR clustering (39). MuSK–LRP4 has been proved to be also involved in the presynaptic differentiation of the NMJ since motoneuronal axons in MuSK^−/−^ or LRP4^−/−^ mutants reach the muscle but fail to stop and innervate the central region of the muscle and to form synaptic vesicles (25, 26). Recently, it was shown that muscle LRP4 aggregates might serve as a retrograde signal and that LRP4 binds to motor axons and induces clustering of synaptic vesicle and active zone proteins (40, 41). Finally, the MuSK–LRP4 complex is also important for the maturation and maintenance of the NMJ (42, 43, 44). Other proteins such as biglycan, amyloid precursor protein, and connective tissue growth factor (CCN2) have been recently identified to interact with MuSK–LRP4 (45, 46, 47, 48). Thus, MuSK–LRP4 constitutes a crucial signaling platform whose activity is regulated by multiple ligands, including agrin, biglycan, Wnts, amyloid precursor protein, and also possibly ColQ.

To better understand how ColQ exerts its different functions and cooperates with the different MuSK–LRP4 ligands during the formation and the maintenance of the NMJ, it is necessary to clarify how ColQ interacts with the MuSK–LRP4 complex and is inserted at the synaptic basal lamina. Although previous studies have suggested that ColQ interacts with MuSK (9, 16, 17, 18, 49), it is still unclear whether this interaction is direct or depends on other partners. It is also not known whether ColQ interacts with LRP4. In addition, it remains unknown whether ColQ modulates MuSK–LRP4 activation. This is an important issue, considering that ColQ might modulate the binding and/or functional activities of the other ligands of the MuSK–LRP4 complex. Using different biochemical and biophysical approaches, we provide evidences that ColQ binds directly to LRP4 and that ColQ–MuSK interaction is indirect and mediated by LRP4. Moreover, we show that the N-terminal region of LRP4, which contains the agrin-binding sites, is also crucial for ColQ–LRP4 interaction. Importantly, we also report that ColQ regulates agrin-induced MuSK phosphorylation and activation. Collectively, our results identify LRP4 as a major receptor for ColQ and bring new insights into the molecular mechanisms of ColQ structural and signaling functions at the NMJ.

## Results

### ColQ and LRP4 interact in transfected heterologous cells and myotubes

**Figure 1:**
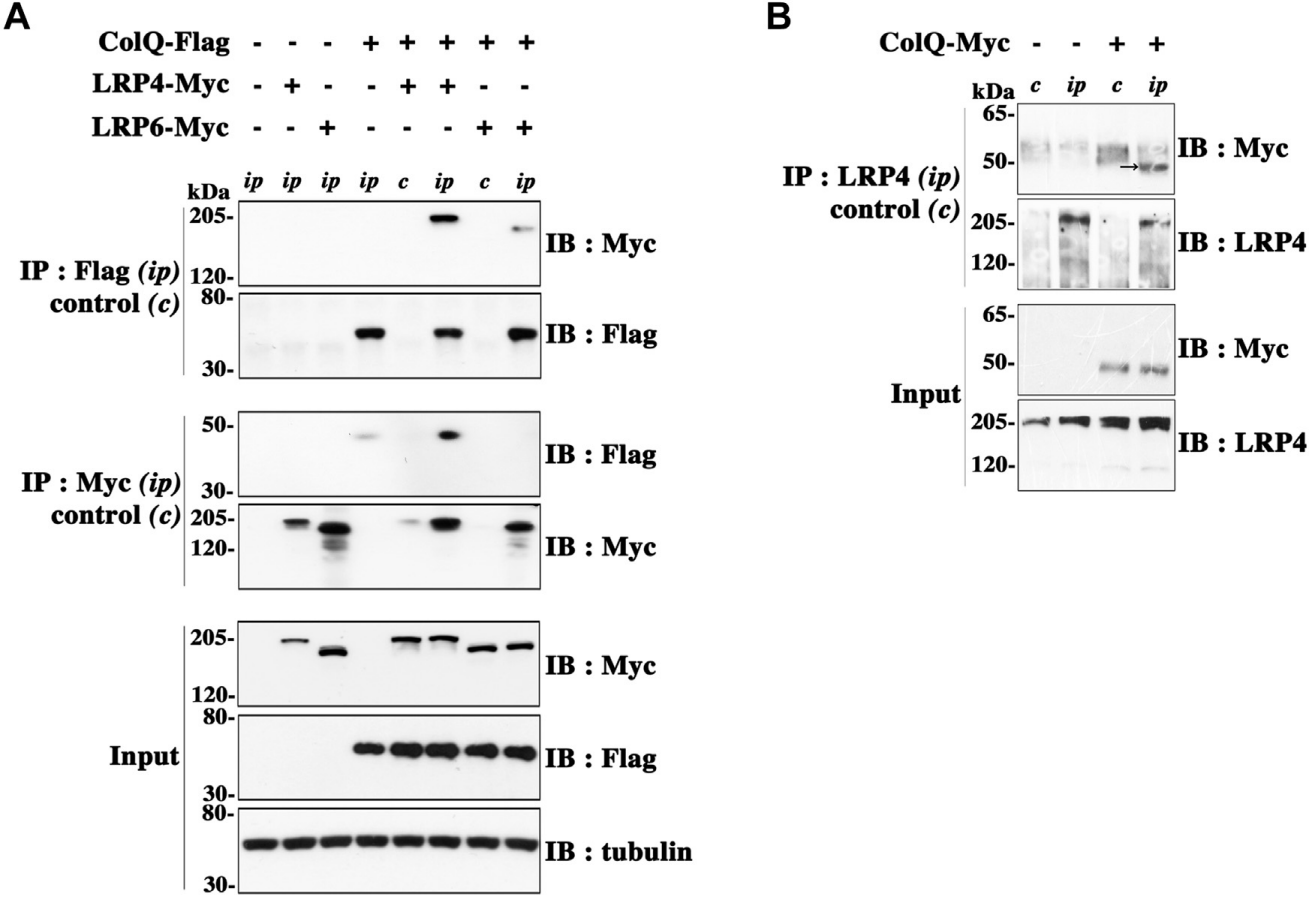
**ColQ and LRP4 coimmunoprecipitate in lysates from transfected heterologous cells and myotubes.***A*, HEK 293T cells were untransfected, cotransfected with either ColQ-Flag and LRP4-Myc or ColQ-Flag and LRP6-Myc, or transfected with each of these constructs alone. Immunoprecipitations were performed with anti-Flag antibodies for ColQ-Flag (*ip*) or with control nonimmune IgGs of the same isotype (*c*), and coprecipitates of LRP4-Myc or LRP6-Myc were analyzed by Western immunoblot using anti-Myc antibodies. After stripping, blots were also probed for Flag to ensure that equal amounts of ColQ-Flag were precipitated. Conversely, LRP4 and LRP6 were precipitated with anti-Myc antibodies (*ip*), and coprecipitated ColQ was detected with anti-Flag antibodies. ColQ clearly coprecipitated with LRP4 but not or very little with LRP6. Cell lysates (*input*) show similar expression of ColQ or of LRP4 and LRP6 in the different conditions. One Western immunoblot image representative of four independent experiments is shown. *B*, ColQ and LRP4 interact in myotubes. ColQ-Myc was expressed (+) or not (−) in ColQ^−/−^ myotubes by viral infection. Immunoprecipitation was performed with polyclonal anti-LRP4 antibodies (*ip*) or control nonimmune IgGs (*c*), and coprecipitates of ColQ-Myc were analyzed by Western immunoblot using anti-Myc antibodies. ColQ-Myc coprecipitated with endogenous LRP4; n = 4. The *arrow* shows the band for ColQ at the expected molecular weight just below 50 kDa. This band is clearly absent and distinct from the diffuse nonspecific signal detected in the controls. ColQ, collagen Q; HEK, human embryonic kidney cell line; IgG, immunoglobulin G; LRP4, low-density lipoprotein receptor–related protein 4.

First, to test a possible interaction between ColQ and LRP4, we cotransfected human embryonic kidney (HEK) 293T cells with complementary DNAs (cDNAs) encoding rat ColQ-Flag and LRP4-Myc. About 48 h after transfection, cells were lysed, and the cell lysates were subjected to immunoprecipitation for ColQ with anti-Flag antibodies followed by Western immunoblotting for LRP4 and ColQ with anti-Myc tag and anti-Flag antibodies, respectively. We observed that LRP4 clearly coprecipitated with ColQ (Fig. 1*A*), suggesting that the two proteins interact in heterologous cells. No signal was obtained in the absence of anti-Flag antibodies, ensuring that there was no nonspecific binding to the beads used for immunoprecipitation. To test the specificity of ColQ–LRP4 interaction, we used LRP6, a homologous member of the LRP family whose structural organization resembles that of LRP4. Cells were cotransfected with ColQ-Flag and LRP6-Myc, and same experiment as aforementioned was performed. Compared with LRP4, only very little LRP6 coprecipitated with ColQ, although LRP4 and LRP6 were expressed at the same levels and the level of ColQ expression was the same in the different conditions (Fig. 1*A*). Reciprocally, when the respective cell lysates were subjected to immunoprecipitation for LRP4 or LRP6, we observed that ColQ coprecipitated only with LRP4 and not with LRP6. The results were identical when using COS cells and are not dependent on the heterologous cell system of expression (data not shown). Coimmunoprecipitation between ColQ and LRP4 was also observed when we used ColQ-GFP, ColQ-Myc, and nontagged LRP4 constructs, precluding any nonspecific binding through the Flag or Myc tags (data not shown). These results suggest that ColQ interacts specifically with LRP4.

We then explored whether the interaction between ColQ and LRP4 was also observed in muscle cells. So far, there are no reliable specific antibodies against ColQ. Thus, we decided to infect ColQ^−/−^ myotubes with the recombinant adeno-associated virus 2 (rAAV2)-ColQ-Myc adenovirus to express a Myc-tagged ColQ in myotubes. About 4 days after transduction, cells were lysed, and the cell lysates were subjected to immunoprecipitation for endogenous LRP4 with anti-LRP4 antibodies followed by Western immunoblotting for ColQ-Myc and LRP4 with anti-Myc-Tag and anti-LRP4 antibodies, respectively. We observed that ColQ-Myc coprecipitated with endogenous LRP4 (Fig. 1*B*). No signal was obtained in the absence of anti-LRP4 antibodies, ensuring that there was no nonspecific binding to the beads. We conclude that ColQ and LRP4 also interact in muscle cells.

### ColQ binds directly to the extracellular domain of LRP4

**Figure 2:**
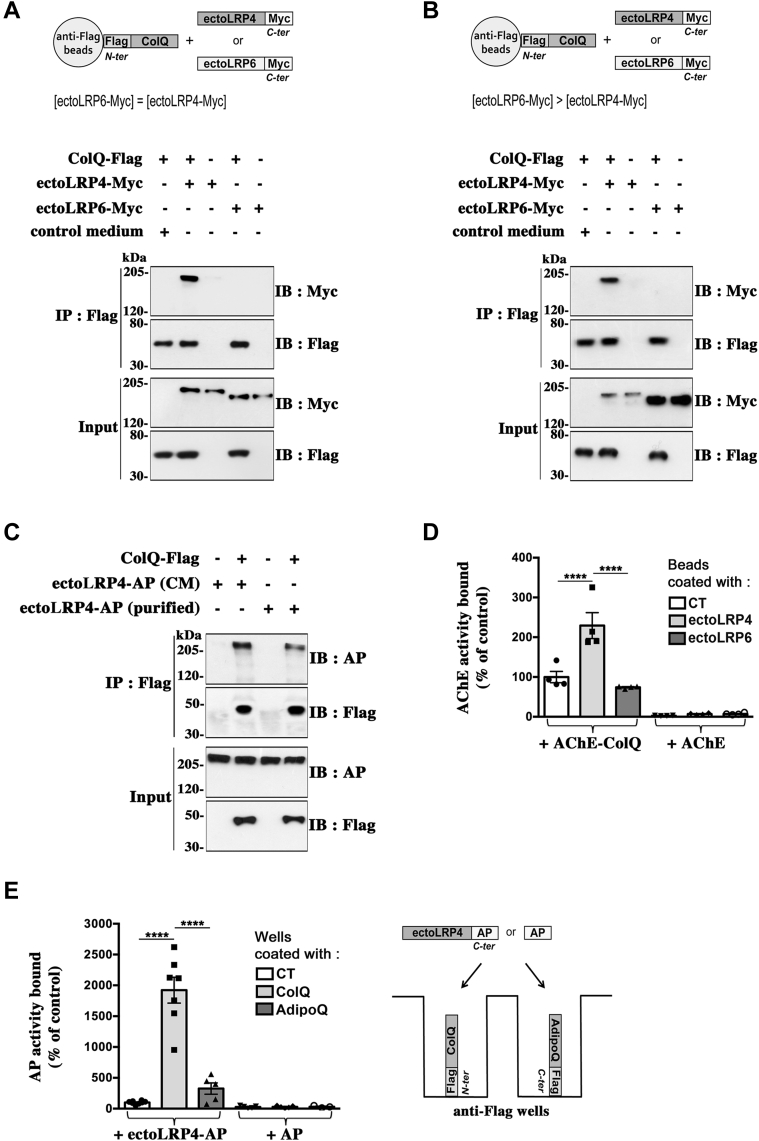
**ColQ binds directly to the extracellular domain of LRP4.***A*, pull-down assays. Magnetic beads were conjugated with ColQ-Flag (+) or not (−) and were subsequently incubated with conditioned media (CM) of HEK 293T cells containing equal amounts of Myc-tagged ectodomain of LRP4 (ectoLRP4-Myc) or LRP6 (ectoLRP6-Myc) or with control medium (HEK 293T cells transfected with an empty vector). Precipitated proteins were analyzed by Western immunoblotting with anti-Myc antibodies. ColQ-Flag interacted with ectoLRP4-Myc but not with ectoLRP6-Myc. Inputs show that comparable amounts of ectoLRP4-Myc and ectoLRP6-Myc were incubated with the ColQ-coated beads; n = 3. *B*, same experiment as in (*A*) except that higher concentrations of ectoLRP6-Myc than ectoLRP4-Myc were tested. Even in these conditions, ColQ bound only to ectoLRP4 and not to ectoLRP6. *C*, uncoated (−) or ColQ-coated (+) beads were incubated with CM expressing ectoLRP4-AP or with ectoLRP4-AP purified from the CM. Purified ectoLRP4-AP bound to ColQ-Flag, indicating a direct interaction between ectoLRP4 and ColQ. *D*, magnetic beads conjugated with equal amounts of ectoLRP4-Myc and ectoLRP6-Myc were incubated with the same amount of enzymatic activity of purified AChE–ColQ (A12 asymmetric forms) or AChE (monomeric globular G1 forms). Bound AChE–ColQ or AChE was quantified by measuring AChE activity. Results are expressed as the mean ± SEM percentage normalized to the control value set as 100% (AChE–ColQ bound to control [CT] uncoated beads). n = 4; ∗∗∗∗*p* < 0.0001, using two-way ANOVA followed by Tukey's multiple comparison post hoc test. For interaction factor: F = 15.99, *p* = 0.0001; for CT *versus* ectoLRP4 *versus* ectoLRP6: F = 16.57, *p* < 0.0001; for AChE–ColQ *versus* AChE: F = 117.5, *p* < 0.0001. AChE–ColQ hetero-oligomers but not monomeric AChE bound to ectoLRP4, confirming a direct interaction between ectoLRP4 and ColQ. *E*, plate-binding assays. Same amounts of ColQ-Flag or AdipoQ-Flag were immobilized on anti-Flag precoated wells before incubation with same concentrations (determined by AP enzymatic activity) of AP or ectoLRP4-AP. Bound AP or ectoLRP4-AP was quantified by measuring AP activity with pNPP as substrate at 405 nm. Results are the mean ± SEM percentage of the control value set as 100% (ectoLRP4-AP bound to CT wells). EctoLRP4-AP on ColQ (n = 7) and AdipoQ (n = 5). AP on ColQ (n = 5) and AdipoQ (n = 3); ∗∗∗∗*p* < 0.0001, using two-way ANOVA followed by Tukey's multiple comparison post hoc test. For interaction factor: F = 35.41, *p* < 0.0001; for CT *versus* ColQ *versus* AdipoQ: F = 35.46, *p* < 0.0001; for ectoLRP4-AP *versus* AP: F = 54.1, *p* < 0.0001. EctoLRP4-AP bound to ColQ but not significantly to AdipoQ, whereas there was no binding of AP to any substrate. AChE, acetylcholinesterase; AP, alkaline phosphatase; ColQ, collagen Q; HEK, human embryonic kidney cell line; LRP4, low-density lipoprotein receptor–related protein 4; pNPP, *p*-nitrophenyl phosphate.

Our results raise the question of whether ColQ, a secreted protein, binds to an intermediary receptor on the cell surface or directly to LRP4. To examine whether ColQ binds directly to the ectodomain of LRP4, we used two different *in vitro* binding assays: a pull-down assay using beads in solution and a modified ELISA as plate-binding assay. For the pull-down assay, ColQ-Flag was immobilized on magnetic beads, which were subsequently incubated with conditioned media of HEK 293T cells containing the same amounts of secreted Myc-tagged ectodomain of either LRP4 (ectoLRP4-Myc) or LRP6 (ectoLRP6-Myc). As shown in Figure 2*A*, ectoLRP4-Myc, but not ectoLRP6-Myc, was precipitated with the ColQ-coated beads, suggesting a specific and direct interaction of ColQ with the ectodomain of LRP4. No binding was observed with control beads. Even when much higher concentrations of ectoLRP6-Myc were incubated with the ColQ-coated beads, ectoLRP6-Myc was not pulled down (Fig. 2*B*). Moreover, purified ectoLRP4-alkaline phosphatase (AP) was also precipitated with ColQ-coated beads, indicating a direct interaction between ColQ and ectoLRP4 (Fig. 2*C*). Same results were obtained with purified ectoLRP4-Myc (not shown). Conversely, we performed assays where magnetic beads conjugated with equal amounts of ectoLRP4-Myc or ectoLRP6-myc were incubated with the same AChE enzymatic activity (0.25 Ellman unit) of sucrose gradient–purified AChE–ColQ (A12 asymmetric forms) or AChE globular G1 forms (nonassociated with ColQ) as a control. We measured the AChE enzymatic activity bound to the beads and observed that AChE–ColQ but not AChE alone binds to ectoLRP4, indicating a direct interaction between ectoLRP4 and ColQ (Fig. 2*D*). No binding of AChE–ColQ to ectoLRP6 was observed.

We then performed plate-binding assays where ColQ-Flag was immobilized on anti-Flag-precoated wells and incubated with conditioned media of HEK 293T cells containing the same concentrations, determined by the same level of AP activity, of AP-conjugated ectodomain of LRP4 (ectoLRP4-AP) or AP. As shown in Figure 2*E*, ectoLRP4-AP, but not AP, bound to ColQ. No AP binding was observed even when adding it at a 10 times higher concentration (not shown), confirming the specific and direct interaction of ColQ with the ectodomain of LRP4 observed in the pull-down assay. Same results were obtained when the wells were coated with purified A12 forms of AChE–ColQ-Flag (data not shown). As a further control, we compared the binding of ectoLRP4-AP to wells that were coated with same amounts of ColQ-Flag or Flag-tagged adiponectin (AdipoQ-Flag), a protein with structural similarities and with the same size range as ColQ. Adiponectin contains a collagenous domain that assembles in triple helix to form homotrimers like ColQ. There was no significant binding of ectoLRP4-AP to AdipoQ, even when more AdipoQ than ColQ was immobilized on anti-Flag wells, indicating that LRP4 binds selectively to ColQ and not to any collagen domain–containing protein (Fig. 2*E*).

**Figure 3:**
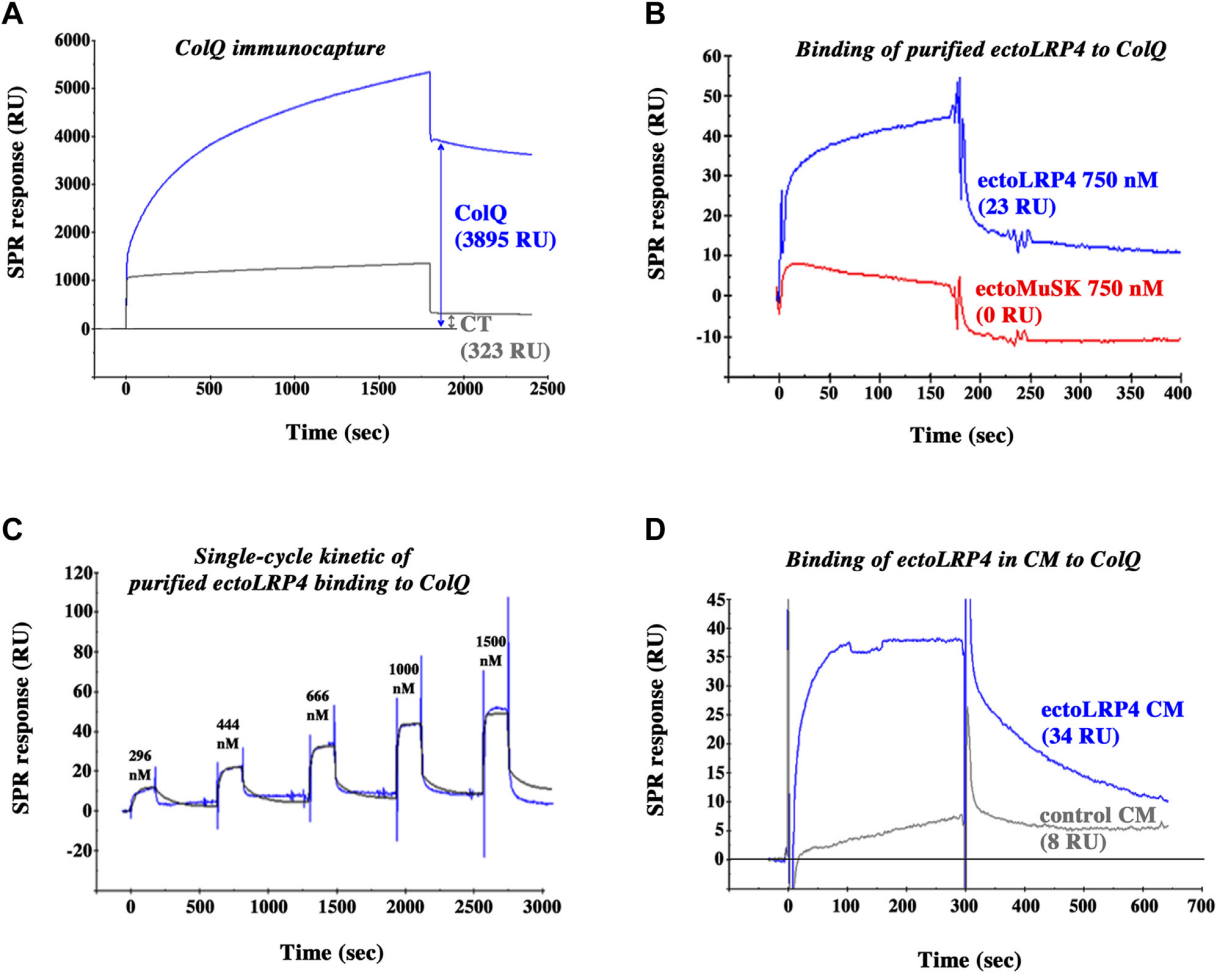
**Surface plasmon resonance analysis of ColQ–LRP4 interaction.***A*, sensorgram of the binding of ColQ-Flag to a sensor chip covalently coated with anti-Flag antibodies to obtain a sensor surface covered with ColQ. Binding responses were measured using report points 30 s after the beginning of the dissociation phase and are indicated in RUs (resonance units). The level of immobilized ColQ reached about 3900 RU. Reference surface was obtained by injecting preparations from untransfected cells (CT) at the same protein concentration as ColQ-Flag preparations. *B*, 750 nM of purified ectoLRP4 (*blue curve*) or purified ectoMuSK (*red curve*) were passed over the ColQ-coated and reference surfaces. Sensorgrams represent the differential specific binding to ColQ after subtraction of the signals obtained on the reference surface. In contrast to ectoLRP4 (binding response of 23 RU), there was no binding of ectoMuSK to ColQ. *C*, sensorgram of the single-cycle kinetics. Five increasing (1.5-fold) concentrations (296, 444, 666, 1000, and 1500 nM) of purified ectoLRP4 were sequentially loaded on the ColQ-coated or reference surfaces without any regeneration step. Reference surface and drift signals were subtracted to obtain the accurate binding profiles. The *black curve* overlaid on the experimental data (*blue curve*) was obtained by fitting the binding profiles to a 1:1 binding model with drifting baseline. The association (*k*_on_) and dissociation (*k*_off_) constants were 9.64 ± 2.86 × 10^4^ M^−1^ s^−1^ and 9.1 ± 2.89 × 10^−3^ s^−1^, respectively, corresponding to a *K*_*d*_ of 9.4 ± 0.19 × 10^−8^ M (n = 2). *D*, conditioned medium containing ectoLRP4-AP at 60 nM (ectoLRP4 CM) or a same dilution of a control conditioned medium (control CM) lacking ectoLRP4-AP, but containing AP at the same concentration, was injected over ColQ-coated and reference surfaces. Sensorgrams display the differential specific binding to ColQ after subtraction of the signals obtained with the reference surface. The binding response for ectoLRP4 CM was substantially higher than for control CM (34 RU *versus* eight RU), revealing a specific binding of ectoLRP4 to ColQ. AP, alkaline phosphatase; CM, conditioned medium; ColQ, collagen Q; LRP4, low-density lipoprotein receptor–related protein 4; MuSK, muscle-specific kinase; RU, resonance unit.

We then used surface plasmon resonance (SPR) technique to study the dynamics of ColQ–LRP4 interaction in real time and to estimate the binding affinity. ColQ-Flag was immobilized by immunocapture (about 3900 resonance units [RUs] for the experiment shown) on a sensor surface to which anti-Flag antibodies had previously been covalently linked (as described in the Experimental procedures section) (Fig. 3*A*). Reference sensor surfaces were obtained using the same procedure by injecting cleared cell lysate solutions from untransfected cells (CT) at the same protein concentration as our ColQ-Flag preparations. In comparison, the sensorgram shows a limited nonspecific binding (about 320 RUs) of factors present in the CT cell lysates to the anti-Flag-coated surface (Fig. 3*A*). First, we assessed the binding of purified recombinant ectoLRP4-His (R&D Systems), which was passed over the ColQ-bound or the reference sensor surfaces. The SPR signal recorded on the reference surface was low (<5 RUs for ectoLRP4 tested at 750 nM) and was subtracted from the signal recorded on the ColQ-bound surface to obtain the response corresponding to the specific binding to ColQ. As shown in Figure 3*B* we observed a specific binding signal of purified ectoLRP4 to ColQ (23 RU), confirming a direct interaction between ColQ and LRP4, as suggested by our pull-down results with purified ectoLRP4 in Figure 2*C*. We next evaluated the affinity of LRP4 for ColQ by following a single-cycle kinetic (SCK) SPR approach. Increasing concentrations (1.5-fold) ranging from 296 to 1500 nM were sequentially injected over the ColQ-bound or reference surfaces without any regeneration step. Figure 3*C* shows a sensorgram of the differential specific binding of ectoLRP4 to ColQ where, for each concentration, the reference and drift signals were subtracted. Binding curves were fitted to a 1:1 binding model with drifting baseline from which an equilibrium dissociation constant (*K*_*d*_) of 9.4 ± 0.19 × 10^−8^ M was derived. In another series of experiments, we also tested the binding of nonpurified ectoLRP4-AP in conditioned medium (CM) that we used in our other binding experiments. CM containing ectoLRP4-AP at 60 nM (ectoLRP4 CM) was loaded on the ColQ-bound or the reference sensor surfaces. In parallel, control CM (lacking ectoLRP4-AP but containing AP at the same concentration) was injected at the same dilution as ectoLRP4 CM. After subtraction of the reference signals, the binding response for ectoLRP4 CM was substantially higher than for control CM (34 RUs *versus* 8 RUs), revealing a specific binding of ectoLRP4 to ColQ (Fig. 3*D*). Consistent with our pull-down and plate-binding assays, the binding occurred at lower concentrations than for purified ectoLRP4, suggesting that factors present in the CM may stabilize the interaction between ectoLRP4 and ColQ. The low binding response for the control CM may be due to perlecan, which is known to be secreted by COS cells (9) and to interact with ColQ (6). Altogether, these results demonstrate that ColQ binds specifically and directly to the extracellular domain of LRP4 with a relatively high affinity.

### Comparison of ColQ–LRP4 and ColQ–MuSK interactions

**Figure 4:**
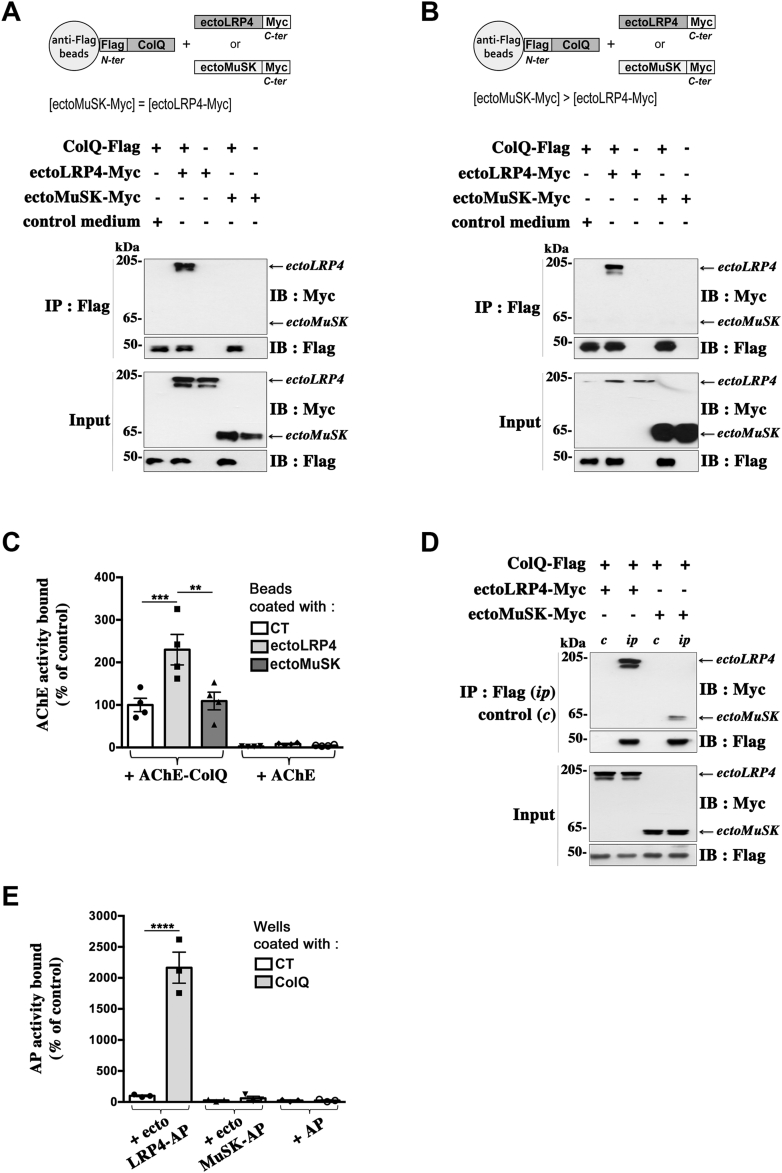
**Comparison of ColQ binding to LRP4 and to MuSK.***A*, pull-down assays. Magnetic beads conjugated with ColQ-Flag (+) or not (−) were incubated with the same amounts of ectoLRP4-Myc or ectoMuSK-Myc as shown in the input. EctoLRP4-Myc but not ectoMuSK-Myc precipitated with ColQ-Flag; n = 3. *B*, same experiment as in (*A*) except that ColQ-coated beads were incubated with higher ectoMuSK-Myc than ectoLRP4-Myc concentrations. *C*, magnetic beads conjugated with equal amounts of ectoLRP4-Myc and ectoMuSK-Myc were incubated with the same amount of enzymatic activity of purified AChE–ColQ or AChE. Bound AChE–ColQ or AChE was quantified by measuring AChE activity. Results are expressed as the mean ± SEM percentage of the control value set as 100% (AChE–ColQ bound to CT ColQ-free beads). n = 4; ∗∗*p* < 0.01; ∗∗∗*p* < 0.001, using two-way ANOVA followed by Tukey's multiple comparison post hoc test. For interaction factor: F = 7.41, *p* = 0.0045; for CT *versus* ectoLRP4 *versus* ectoMuSK: F = 8.63, *p* = 0.0024; for AChE–ColQ *versus* AChE: F = 91.22, *p* < 0.0001. No significant binding of AChE–ColQ to ectoMuSK was detected, whereas AChE–ColQ bound to ectoLRP4. *D*, coimmunoprecipitation experiments. HEK 293T cells were cotransfected with either ColQ-Flag and ectoLRP4-Myc or ColQ-Flag and ectoMuSK-Myc. Immunoprecipitations were performed with anti-Flag antibodies (*ip*) or control nonimmune IgGs (*c*), and immunoprecipitates were revealed by Western immunoblot using anti-Myc and anti-Flag antibodies. Lysates (*input*) were analyzed by Western immunoblot to show similar expression of ectoLRP4 and ectoMuSK in the different conditions. ColQ expression was constant between the different conditions. Much less ectoMuSK was coprecipitated with ColQ than ectoLRP4. *E*, plate-binding assays. ColQ-Flag was immobilized on anti-Flag precoated wells before incubation with same concentrations of AP, ectoLRP4-AP, or ectoMuSK-AP. Bound proteins to ColQ-coated or control (CT) wells were quantified by measuring AP activity. Results are expressed as the mean ± SEM percentage of the control value set as 100% (ectoLRP4-AP bound to CT wells). n = 3; ∗∗∗∗*p* < 0.0001, using two-way ANOVA followed by Tukey's multiple comparison post hoc test. For interaction factor: F = 65.18, *p* < 0.0001; for CT *versus* ColQ: F = 68.48, *p* < 0.0001; for ectoLRP4-AP *versus* ectoMuSK-AP *versus* AP: F = 75.1, *p* < 0.0001. In contrast to ectoLRP4-AP, there was no significant binding of ectoMuSK-AP to ColQ. AChE, acetylcholinesterase; AP, alkaline phosphatase; ColQ, collagen Q; HEK, human embryonic kidney cell line; IgG, immunoglobulin G; LRP4, low-density lipoprotein receptor–related protein 4; MuSK, muscle-specific kinase.

Previous studies have suggested that ColQ, through its C-terminal domain, is able to bind to MuSK (9, 16, 17). Thus, we decided to compare ColQ–LRP4 and ColQ–MuSK interactions by analyzing first the respective binding of ectoMuSK-Myc (secreted Myc-tagged ectodomain of MuSK) and ectoLRP4-Myc to ColQ using our pull-down assay. To this end, ColQ-coated beads were incubated with conditioned media of HEK 293T cells containing the same amounts of ectoMuSK-Myc or ectoLRP4-Myc. Surprisingly, in contrast to ectoLRP4-Myc, ectoMuSK-Myc was not pulled down with ColQ-coated beads (Fig. 4*A*), even when the ColQ-coated beads were incubated with much higher concentrations of ectoMuSK-Myc (Fig. 4*B*). No interaction with ColQ was detected either, when the same pull-down experiments were performed with purified ectoMuSK-Myc (data not shown). Conversely, we performed pull-down assays where magnetic beads conjugated with equal amounts of ectoMuSK-Myc or ectoLRP4-myc were incubated with 0.25 Ellman unit of purified AChE–ColQ or AChE globular G1 forms as a control. Consistent with our aforementioned results, we failed to detect any significant binding of AChE–ColQ to ectoMuSK whereas AChE–ColQ bound to ectoLRP4 (Fig. 4*C*). We used the ectodomain of rat MuSK, which has been used in previous studies (37) so that it is quite unlikely that the failure to observe any binding of ectoMuSK to ColQ is due to misfolding of the ectodomain. In addition, to verify whether our ectoMuSK-Myc construct was able to interact with ColQ in a cellular context as observed by others (18) and as observed for full-length MuSK (9, 17), we evaluated the coimmunoprecipitation between ColQ and ectoMuSK in HEK 293T cells cotransfected with cDNAs encoding ColQ-Flag and ectoMuSK-Myc. As shown in Figure 4*D*, ectoMuSK-Myc was coprecipitated with ColQ-Flag. We also compared ColQ-ectoMuSK and ColQ-ectoLRP4 coimmunoprecipitations in extracts from HEK 293T cells, which had been cotransfected with either ColQ-Flag and ectoLRP4-Myc or ColQ-Flag and ectoMuSK-Myc, so as to obtain similar expression levels of ectoLRP4-Myc and ectoMuSK-Myc. Interestingly, we observed that much less ectoMuSK was coprecipitated with ColQ than ectoLRP4 (Fig. 4*D*). In addition, we also used our plate-binding assay to compare the binding of ectoLRP4-AP and ectoMuSK-AP added to ColQ-coated wells at the same concentrations. In contrast to ectoLRP4-AP, there was no binding of ectoMuSK-AP to ColQ (Fig. 4*E*), even when added at a six times higher concentration than ectoLRP4-AP (not shown), confirming the above pull-down results. Moreover, our SPR experiments showed that, in contrast to ectoLRP4, there was no binding to ColQ of the purified recombinant ectoMuSK (R&D Systems) tested at the same high concentration of 750 nM as the purified ectoLRP4 (Fig. 3*B*). Together, these data suggest that the interaction between ColQ and MuSK is indirect or of a very low affinity and unstable in our *in vitro*–binding experimental conditions.

### LRP4 potentiates the interaction of ColQ with MuSK

**Figure 5:**
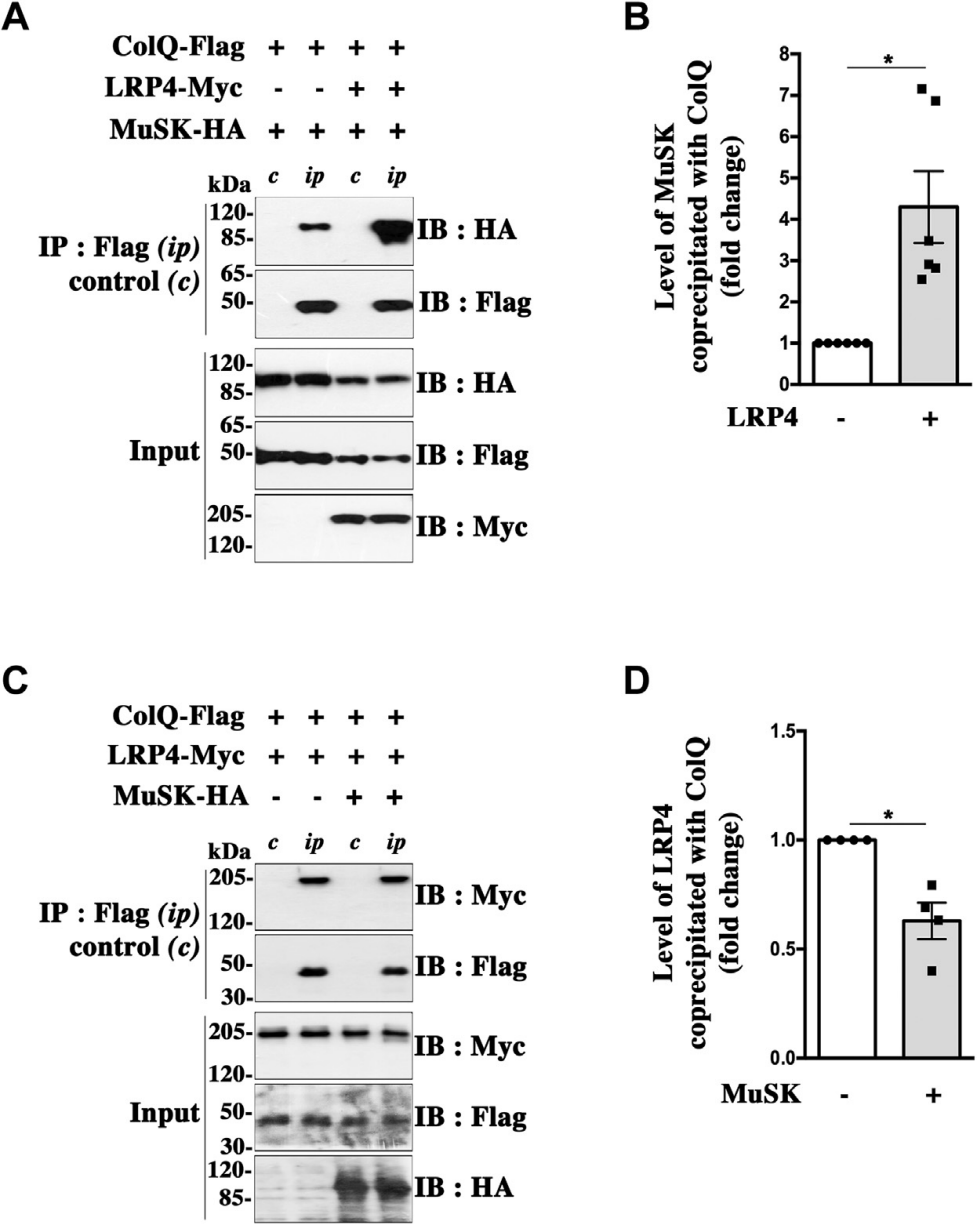
**LRP4 potentiates ColQ–MuSK interaction.***A*, influence of LRP4 on ColQ–MuSK coimmunoprecipitation. HEK 293T cells were cotransfected with ColQ-Flag and MuSK-HA in the presence (+) or the absence (−) of cDNAs encoding LRP4-Myc. ColQ was immunoprecipitated (*ip*) with anti-Flag antibodies, and coprecipitated MuSK-HA was revealed using anti-HA antibodies. Control experiments (*c*) without adding anti-Flag antibodies to the cell lysates were performed in parallel. *B*, quantification of data in (*A*). The different band intensities were measured using ImageJ software. MuSK-HA signals were normalized to those of precipitated ColQ. The levels of MuSK coprecipitated with ColQ are expressed as the mean ± SEM fold change relative to the condition without LRP4 (n = 6; ∗*p* < 0.05, using one-sample *t* test). In the presence of LRP4, the level of MuSK coprecipitated with ColQ was increased by a factor of about fourfold. *C*, influence of MuSK on ColQ–LRP4 coimmunoprecipitation. Cells were cotransfected with ColQ-Flag and LRP4-Myc in the presence (+) or not (−) of MuSK-HA. ColQ was immunoprecipitated as aforementioned, and coprecipitated LRP4-Myc was evaluated by Western immunoblot. *D*, quantification of data in (*C*). Results are the mean ± SEM fold change relative to the condition without MuSK. n = 4; ∗*p* < 0.05. cDNA, complementary DNA; ColQ, collagen Q; HEK, human embryonic kidney cell line; LRP4, low-density lipoprotein receptor–related protein 4; MuSK, muscle-specific kinase.

We next investigated whether LRP4 may potentiate ColQ–MuSK interaction. For this purpose, we cotransfected HEK 293T cells with ColQ-Flag and MuSK-HA constructs in the presence or not of cDNAs encoding LRP4-Myc. About 48 h after transfection, cells were lysed and the cell lysates were subjected to immunoprecipitation for ColQ. We observed that significantly more (about 4-fold) MuSK is coprecipitated with ColQ in the presence of LRP4, indicating a potentiation of ColQ–MuSK interaction by LRP4 (Fig. 5, *A* and *B*). When LRP4 was cotransfected, ColQ and MuSK expression were reduced at similar levels with an unchanged MuSK–ColQ ratio, which indicates that the LRP4-induced increase of ColQ–MuSK interaction is not linked to any increase of ColQ or MuSK expression. These results suggest that LRP4, by interacting together with ColQ and MuSK, may stabilize an interaction of low affinity between ColQ and MuSK within a ternary complex, and/or serve as an intermediate link between these two proteins enabling an indirect interaction. Alternatively, it is possible that LRP4 modifies the conformation of MuSK and/or ColQ favoring their interaction. In contrast, when HEK 293T cells were cotransfected with ColQ-Flag and LRP4-Myc in the presence or not of cDNAs encoding MuSK-HA, we observed that ColQ–LRP4 coprecipitation was slightly reduced by the presence of cotransfected MuSK (Fig. 5, *C* and *D*).

### Role of the ColQ C-terminal domain in the ColQ–LRP4 interaction

**Figure 6:**
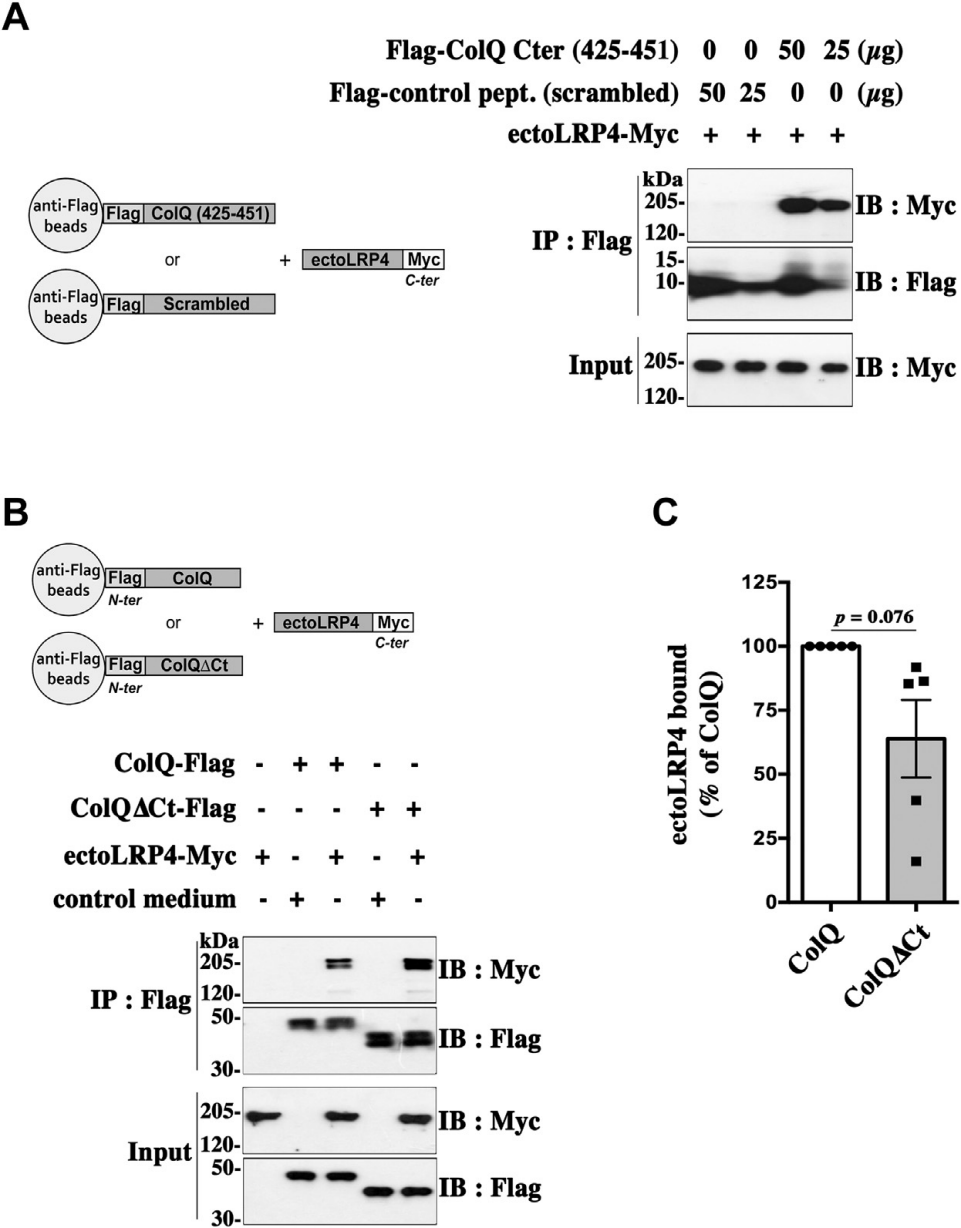
**Role of the ColQ C-terminal domain in the ColQ–LRP4 interaction.***A*, 25 or 50 μg of a Flag-tagged peptide corresponding to the last 27 amino acids of the ColQ C-terminal domain (Flag-ColQ Cter [425–451]) or of a Flag-control peptide corresponding to the scrambled 425 to 451 sequence were used to coat magnetic beads, which were subsequently incubated with purified ectoLRP4-Myc. Pulled down ectoLRP4-Myc was analyzed by Western immunoblot with anti-Myc antibodies. EctoLRP4-Myc interacts with the Flag-ColQ Cter peptide but not with the corresponding scrambled peptide. The image is representative of three independent experiments. *B*, magnetic beads coated with similar amounts of ColQ-Flag and ColQΔCt-Flag or ColQ-free beads as a control were incubated with conditioned media of HEK 293T cells containing equal amounts of ectoLRP4-Myc, as shown in inputs, or with control medium. *C*, quantification of ectoLRP4 bound to ColQ or ColQΔCt from data in (*B*) reveals a lower binding of ectoLRP4 to ColQΔCt than to ColQ, albeit statistically not significant. Results were normalized to precipitated ColQ or ColQΔCt and are expressed as the mean ± SEM of ColQ condition set as 100%; n = 5. ColQ, collagen Q; HEK, human embryonic kidney cell line; LRP4, low-density lipoprotein receptor–related protein 4.

Because the C-terminal domain of ColQ, which does not interact with perlecan, is essential for AChE–ColQ anchoring at the NMJ (9, 10), we explored whether this domain is involved in the interaction between ColQ and LRP4. Therefore, we decided to produce a Flag-tagged peptide corresponding to the last 27 amino acids of the C-terminal domain of rat ColQ (Flag-ColQ Cter [amino acids [aa] 425–451]) and a control peptide corresponding to the scrambled 425 to 451 sequence. Same amounts of these peptides were immobilized on magnetic beads, which were subsequently incubated with equal amounts of purified ectoLRP4-Myc. As shown in Figure 6*A*, ectoLRP4-Myc was precipitated with Flag-ColQ Cter (aa 425–451)-coated beads but not with the control peptide-coated beads, indicating that the C terminus end of ColQ is able to bind to LRP4. Then, to establish whether the C terminus of ColQ is the single domain responsible for ColQ binding to LRP4, we generated a Flag-tagged ColQ construct where the C-terminal sequence from amino acid 375 to 451 (end) was deleted (ColQΔCt-Flag). Magnetic beads coated with equivalent amounts of ColQ-Flag and ColQΔCt-Flag were incubated with conditioned media of HEK 293T cells containing same amounts of ectoLRP4-Myc (Fig. 6*B*). After normalization of the results to the amount of ColQ-Flag and ColQΔCt-Flag actually linked to the beads, we observed a lower binding of ectoLRP4-Myc to ColQΔCt-Flag than to ColQ-Flag, albeit statistically not significant, suggesting a possible role of the ColQ C terminus in ColQ–LRP4 interaction (Fig. 6*C*). However, the binding was clearly not completely abolished, indicating the involvement of other ColQ domains in ColQ–LRP4 interaction. Together, these results suggest that ColQ–LRP4 interaction involves the C-terminal as well as other domains of ColQ.

### Domains of LRP4 interacting with ColQ

**Figure 7:**
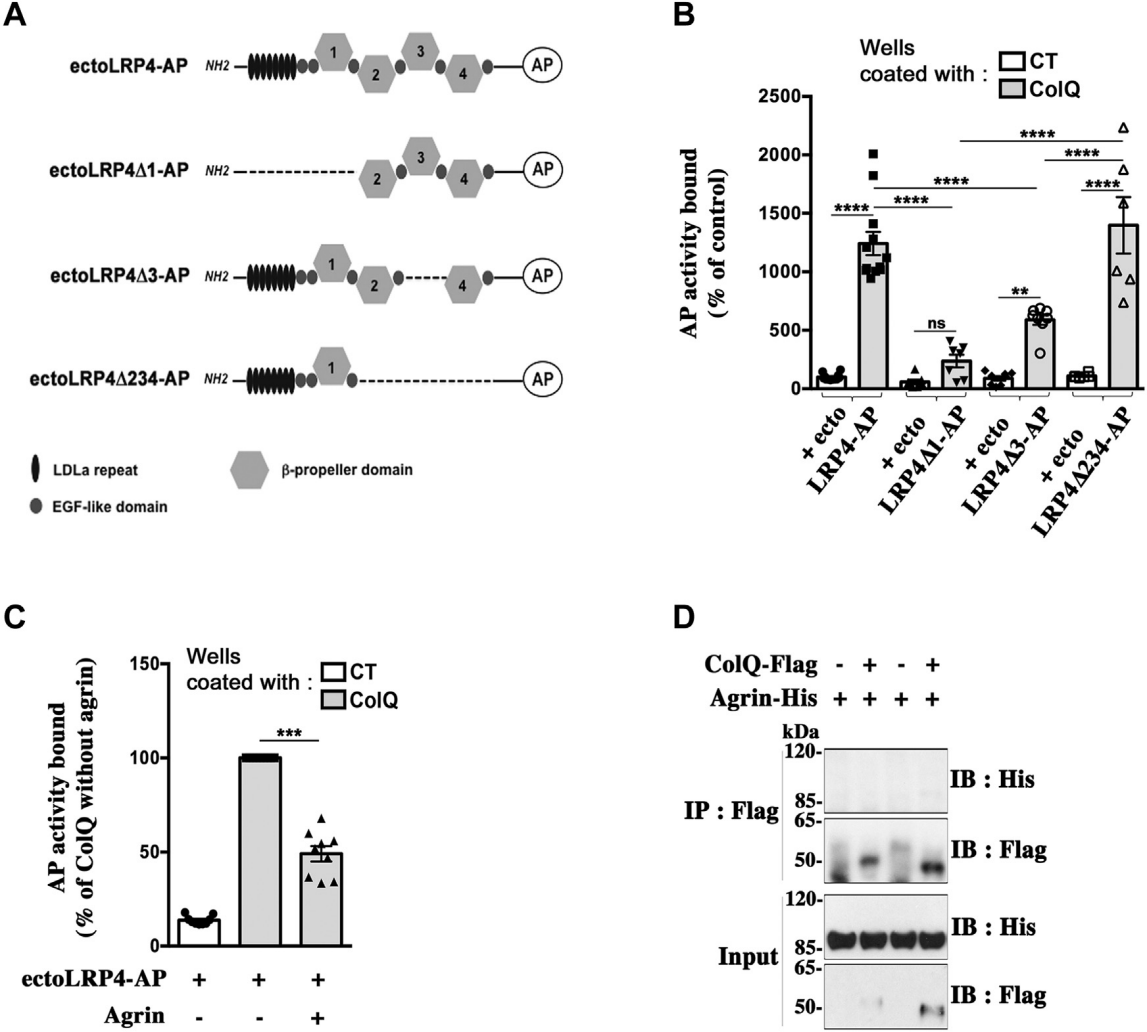
**Domains of LRP4 interacting with ColQ.***A*, schematic representation of ectoLRP4-AP and its deletion mutants. *B*, plate-binding assays. Same concentrations of ectoLRP4-AP and of the indicated deletion mutants were added to ColQ-coated or CT wells. Bound proteins were quantified by measuring AP activity. Results are expressed as the mean ± SEM percentage of the control value set as 100% (ectoLRP4-AP bound to CT wells). n ≥ 6; ∗∗*p* < 0.01; ∗∗∗∗*p* < 0.0001, using two-way ANOVA followed by Tukey's multiple comparison post hoc test. For interaction factor: F = 18.07, *p* < 0.0001; for CT *versus* ColQ: F = 162, *p* < 0.0001; for the comparison of the different ectoLRP4 mutants: F = 21.01, *p* < 0.0001. The N-terminal region of LRP4 plays a crucial role in ColQ–LRP4 interaction as its deletion (ectoLRP4Δ1-AP) compromised binding to ColQ. Conversely, the N-terminal region alone (ectoLRP4Δ234-AP) bound to ColQ at the same level as ectoLRP4-AP. *C*, ColQ-coated wells were incubated with 25 nM of ectoLRP4-AP in the presence or not of 500 nM purified recombinant neural agrin. Results are the mean ± SEM percentage of ectoLRP4-AP bound to ColQ wells in the absence of agrin (set as 100%; n = 9, ∗∗∗*p* < 0.001, using one-sample *t* test). The binding of ecto-LRP4 to ColQ was reduced by more than 50% in the presence of agrin. *D*, pull-down assay where ColQ-coated beads were incubated with 500 nM of His-tagged recombinant neural agrin. Agrin signals were analyzed by Western immunoblot using antibodies against His-tag. No agrin was coprecipitated with ColQ. AP, alkaline phosphatase; ColQ, collagen Q; LRP4, low-density lipoprotein receptor–related protein 2; ns, not significant.

To identify regions in the ectodomain of LRP4 that mediate the association with ColQ, we generated several truncated forms of ectoLRP4-AP (Fig. 7*A*) that were tested for their binding to ColQ using the plate-binding assay described before. Same activity levels of full-length or mutated ectoLRP4-AP were added to immobilized ColQ. We observed that the binding of ectoLRP4Δ1-AP was reduced by more than 80% compared with ectoLRP4-AP, indicating that the N-terminal region, containing the LDLa repeats, the two first EGF-like domains, and the first β-propeller domain, plays a crucial role in ColQ–LRP4 interaction (Fig. 7*B*). Importantly and consistent with this result, the binding of ectoLRP4Δ234-AP, which is composed of only this N-terminal region, was unchanged in comparison to ectoLRP4-AP (Fig. 7*B*), excluding that the loss of interaction between ectoLRP4Δ1-AP and ColQ is due to any conformational change of another remaining domain in the ectoLRP4Δ1 mutant. When only the third β-propeller domain was deleted (ectoLRP4Δ3-AP), the binding to ColQ was reduced by approximately half, suggesting that this region also contributes to the interaction but to a lesser extent. The effect of the third β-propeller domain deletion may be compensated in the ectoLRP4Δ234-AP mutant as the second and/or the fourth β-propeller domains may restrain ColQ binding as observed for LRP4–agrin interaction (50). Since the LRP4 N-terminal region, which is important for ColQ binding, also contains the agrin-binding sites (50), we next examined whether agrin may influence ColQ–LRP4 interaction in our plate-binding assay. ColQ-coated wells were incubated with 25 nM of ectoLRP4-AP in the presence or not of 500 nM neural agrin. The binding of ecto-LRP4 to ColQ was reduced by more than 50% in the presence of agrin (Fig. 7*C*). Importantly, to exclude the possibility that this effect may be due to any binding of agrin to ColQ, we performed a pull-down assay where ColQ-coated beads were incubated with the same concentration of agrin used in the plate-binding assay. As shown in Figure 7*D*, agrin did not bind to ColQ, indicating that the reduction of ColQ–LRP4 interaction by agrin observed in Figure 7*C* indeed results from agrin binding to LRP4. Together, these results indicate that agrin and ColQ both bind to the N-terminal region of LRP4 and suggest that they mutually influence each other for their binding to LRP4.

### ColQ regulates agrin-induced MuSK phosphorylation and AChR clustering

**Figure 8:**
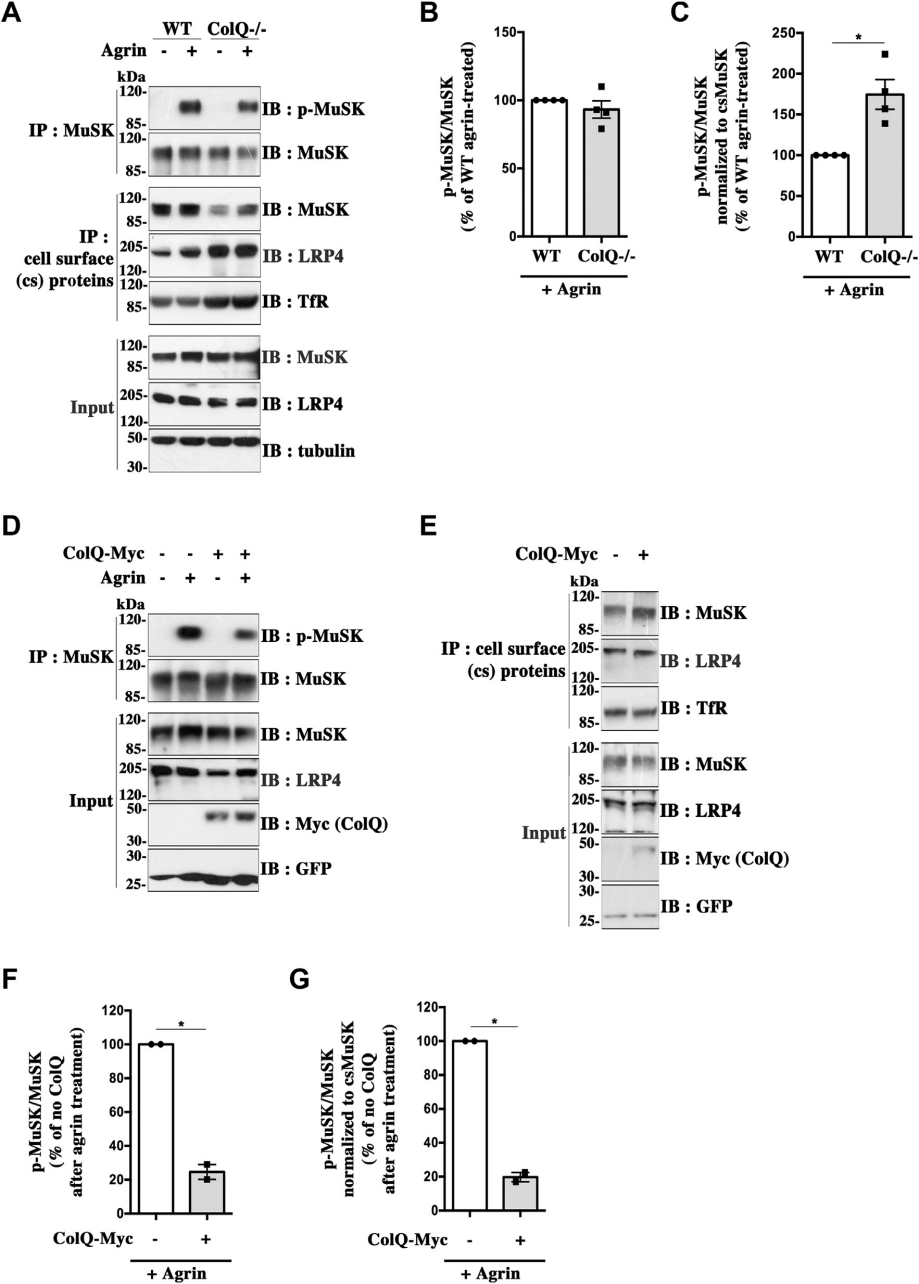
**ColQ regulates agrin-induced MuSK phosphorylation in myotubes.***A*, MuSK phosphorylation was analyzed in WT and ColQ^−/−^ myotubes treated (+) or not (−) with 10 nM of neural agrin for 1 h before cell lysis. MuSK was immunoprecipitated, and the levels of MuSK tyrosine phosphorylation were assessed after Western blot by probing immunoprecipitated MuSK with anti-phosphotyrosine antibody 4G10. Immunoblots were then stripped and reprobed with anti-MuSK antibody to visualize precipitated MuSK. Agrin induced a strong tyrosine phosphorylation of MuSK in WT and ColQ^−/−^ myotubes, whereas no phosphorylation was observed in untreated myotubes. In parallel, cell surface (cs) proteins of myotubes treated in the same way were isolated and probed with antibodies against MuSK or LRP4 to determine their cs levels. Blots were also probed with antibodies against the membrane transferrin receptor (TfR) used as a loading control to normalize the results. The cs levels of MuSK relative to TfR were decreased in the absence of ColQ, whereas those of LRP4 were increased. Total levels of MuSK and LRP4 were unchanged in WT and ColQ^−/−^ myotubes (*input*). *B*, quantification of MuSK phosphorylation signals normalized to precipitated MuSK (p-MuSK/MuSK) for data in (*A*). Results are expressed as the mean ± SEM percentage of agrin-treated WT myotube condition set as 100% (n = 4; ns). *C*, quantification of data in (*A*) where p-MuSK/MuSK ratios were normalized to cs levels of MuSK before agrin treatment. When taking into account the cs levels of MuSK, agrin-induced MuSK phosphorylation level was increased by about 75% in ColQ^−/−^ compared with WT myotubes (n = 4; ∗*p* < 0.05, using one-sample *t* test). *D*, ColQ^−/−^ muscle cells were transduced to express ColQ-Myc (+) or not (−). After differentiation, the myotubes were treated (+) or not (−) with 10 nM of neural agrin for 1 h, and MuSK tyrosine phosphorylation was assessed as aforementioned. *E*, MuSK and LRP4 cs levels of ColQ^−/−^ myotubes re-expressing ColQ-Myc (+) or not (−) and not treated with agrin were analyzed as aforementioned. *F*, quantification of p-MuSK/MuSK ratios for data in (*D*). Results are expressed as the mean ± SEM percentage of the condition corresponding to agrin-treated ColQ^−/−^ myotubes without ColQ-Myc set as 100% (n = 2; ∗*p* < 0.05, using one-sample *t* test). *G*, quantification of data in (*D* and *E*) where p-MuSK/MuSK ratios were normalized to cs levels of MuSK. Agrin-induced MuSK phosphorylation level was decreased by about 82% in ColQ^−/−^ myotubes re-expressing ColQ compared with ColQ-deficient myotubes (n = 2; ∗*p* < 0.05, using one-sample *t* test). ColQ, collagen Q; MuSK, muscle-specific kinase.

Our observations that ColQ binds to the same N-terminal region of LRP4 as agrin together with previous results indicating that ColQ modulates postsynaptic events, such as AChR expression and clustering, which are controlled by MuSK activity (19), suggested that ColQ might regulate agrin-induced MuSK activation. To test this hypothesis, we first analyzed agrin-induced MuSK tyrosine phosphorylation in WT and ColQ-deficient myotubes derived from a stable muscle cell line previously described (9). Cultured differentiated myotubes were treated or not with neural agrin for 1 h, and cell lysates were subjected to immunoprecipitation of MuSK. Resulting precipitates were probed with the anti-phosphotyrosine antibody 4G10. In parallel, cell surface proteins of myotubes treated in the same way were labeled with a membrane-impermeable biotinylation reagent and isolated with streptavidin beads to determine cell surface levels of MuSK or LRP4 before and after agrin treatments. No signal was detected when biotinylation was omitted (not shown). As shown in Figure 8*A*, agrin induced a strong phosphorylation of MuSK in WT and ColQ^−/−^ myotubes, whereas no signal for MuSK phosphorylation was detected in untreated myotubes as expected. The signal for MuSK tyrosine phosphorylation quantified from the total MuSK precipitates was only very slightly reduced in ColQ^−/−^ compared with WT agrin-treated myotubes (93.2 ± 6.3% of WT at 100%, ns; n = 4) (Fig. 8*B*). However, the amounts of MuSK associated to the plasma membrane appeared to be strongly diminished by about 42% in ColQ^−/−^ compared with WT myotubes (57.8 ± 4.9% of WT at 100%, *p* < 0.05, n = 4), whereas total MuSK protein levels remained unchanged (Fig. 8*A*; *input*) in accordance with previous findings (19). This difference in cell surface levels of MuSK between WT and ColQ^−/−^ myotubes remained unchanged after 1 h of agrin treatment. In contrast, the cell surface levels of the agrin receptor LRP4 varied in a opposite way to MuSK as they were increased by 2.1 ± 0.25-fold (*p* < 0.05; n = 4) in ColQ^−/−^ compared with WT myotubes, indicating that any decrease in agrin responsiveness of ColQ^−/−^ myotubes was not attributable to a deficit in LRP4. Considering that agrin acts through MuSK exposed at the cell surface, we normalized phosphorylated MuSK signals to the cell surface levels of MuSK at the time agrin treatment was initiated. Quantitative analysis using this normalization revealed that the level of agrin-induced MuSK phosphorylation is in fact increased by about 75% in ColQ^−/−^ compared with WT myotubes (Fig. 8*C*), although the global tyrosine phosphorylation signal for MuSK is almost unchanged or very slightly reduced in ColQ-deficient myotubes as mentioned previously (Fig. 8*B*).

We then examined whether the aforedescribed increase in agrin-induced MuSK phosphorylation level in ColQ^−/−^ myotubes could be reversed by re-expressing ColQ. To this end, ColQ^−/−^ myotubes were infected with the rAAV2-ColQ-Myc virus to express a Myc-tagged ColQ, whereas control ColQ^−/−^ myotubes were infected with rAAV2-enhanced GFP (EGFP). Four days after transduction, cells were treated or not with 10 nM of neural agrin for 1 h, and MuSK phosphorylation and cell surface levels of MuSK and LRP4 were analyzed as aforementioned. As shown in Figure 8, *D* and *F*, the level of agrin-induced MuSK phosphorylation was strongly reduced by about 75% in ColQ^−/−^ myotubes re-expressing ColQ compared with control ColQ^−/−^ myotubes that were not infected with rAAV2-ColQ-Myc. In addition, MuSK cell surface levels were increased by 1.4 ± 0.12-fold (*p* < 0.05; n = 4) in ColQ^−/−^ myotubes re-expressing ColQ compared with control ColQ^−/−^ myotubes, whereas LRP4 cell surface levels were slightly increased (Fig. 8*E*). When phosphorylated MuSK signals were normalized to cell surface levels of MuSK, the decrease in agrin-induced MuSK phosphorylation reached about 82% in ColQ^−/−^ myotubes re-expressing ColQ compared with control ColQ^−/−^ myotubes (Fig. 8*G*). These results indicate that the alterations in MuSK phosphorylation and cell surface expression in ColQ^−/−^ myotubes are rescued by ColQ re-expression and directly result from the absence of ColQ and not from some indirect effect.

**Figure 9:**
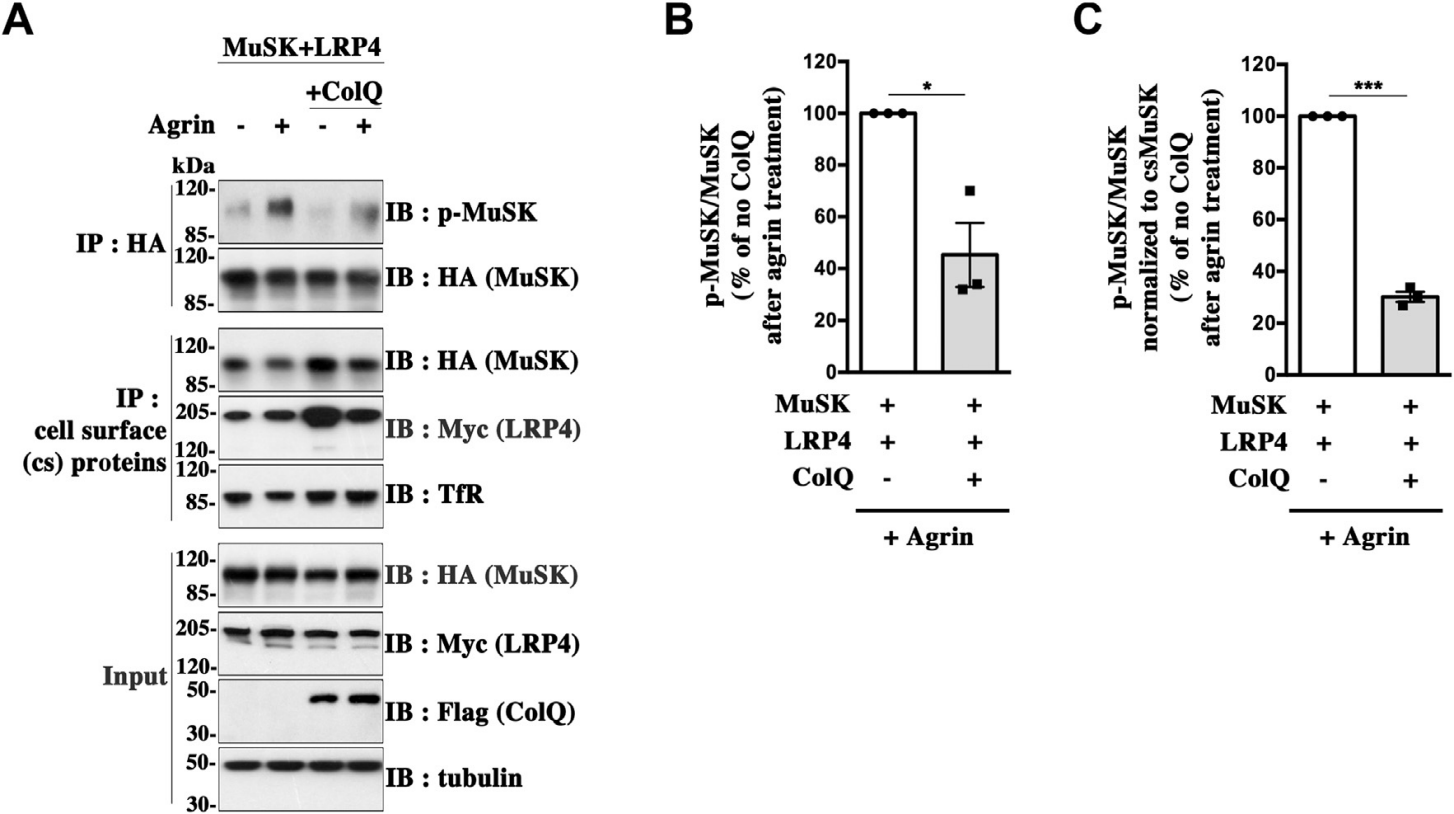
**ColQ regulates agrin-induced MuSK phosphorylation in transfected heterologous cells.***A*, HEK 293T cells were cotransfected with MuSK-HA and LRP4-Myc and in addition with ColQ-Flag or an empty plasmid. After 48 h, the cells were treated or not with 10 nM of neural agrin for 1 h. MuSK was immunoprecipitated, and the levels of MuSK phosphorylation were assessed as aforementioned for myotubes. The blots were stripped and reprobed with anti-HA antibodies to visualize precipitated MuSK. Cell surface (cs) levels of MuSK and LRP4 were analyzed as aforementioned, using anti-HA and anti-Myc antibodies, respectively, for immunodetection. *B*, quantification of the p-MuSK/MuSK ratios for data in (*A*). Results are expressed as the mean ± SEM percentage of agrin-treated HEK cells expressing MuSK and LRP4 but not ColQ (n = 3; ∗*p* < 0.05, using one-sample *t* test). MuSK tyrosine phosphorylation in total MuSK precipitates was decreased by about 50% in the presence of ColQ. *C*, quantification of data in (*A*) where p-MuSK/MuSK ratios were normalized to cs MuSK levels before agrin treatment. When taking into account the cs levels of MuSK, the decrease of agrin-induced MuSK phosphorylation level reached about 67% in the presence of ColQ (n = 3; ∗∗∗*p* < 0.001, using one-sample *t* test). ColQ, collagen Q; HEK, human embryonic kidney cell line; MuSK, muscle-specific kinase.

To further analyze whether ColQ regulation of agrin-induced MuSK–LRP4 activation is direct or not, we decided to test whether ColQ is able to modulate agrin-induced MuSK phosphorylation in HEK 293T cells. We cotransfected ColQ-Flag or an empty plasmid with MuSK-HA and LRP4-Myc in HEK 293T cells. About 48 h later, the cells were treated or not with 10 nM of neural agrin for 1 h before cell lysis. MuSK was isolated by immunoprecipitation and assayed for tyrosine phosphorylation by Western immunoblot using 4G10 antibody. As aforementioned, cell surface levels of MuSK and LRP4 were ascertained in parallel. Agrin stimulated strongly MuSK phosphorylation in HEK cells cotransfected only with MuSK and LRP4 (Fig. 9*A*). In the presence of ColQ, we observed a strong reduction of about 50% of agrin-induced MuSK phosphorylation level (Fig. 9*B*). After normalization to cell surface MuSK signals, the reduction was even more pronounced reaching about 67% (Fig. 9*C*). Importantly, ColQ-induced decrease of MuSK phosphorylation was not attributable to reduced cell surface levels of MuSK or LRP4 in the ColQ-transfected cells, since both cell surface MuSK and LRP4 levels normalized to TfR (transferrin receptor) were slightly increased in the presence of ColQ, whereas total MuSK and LRP4 protein levels were unchanged. Noteworthy, these regulations of MuSK phosphorylation and cell surface levels of MuSK and LRP4 are very similar to those obtained for ColQ^−/−^ myotubes re-expressing ColQ. Together, these results indicate a direct partial inhibitory effect of ColQ on agrin-induced MuSK–LRP4 activation and resulting MuSK phosphorylation level, which is independent of AChE and that does not seem to require any other muscle-specific factor.

**Figure 10:**
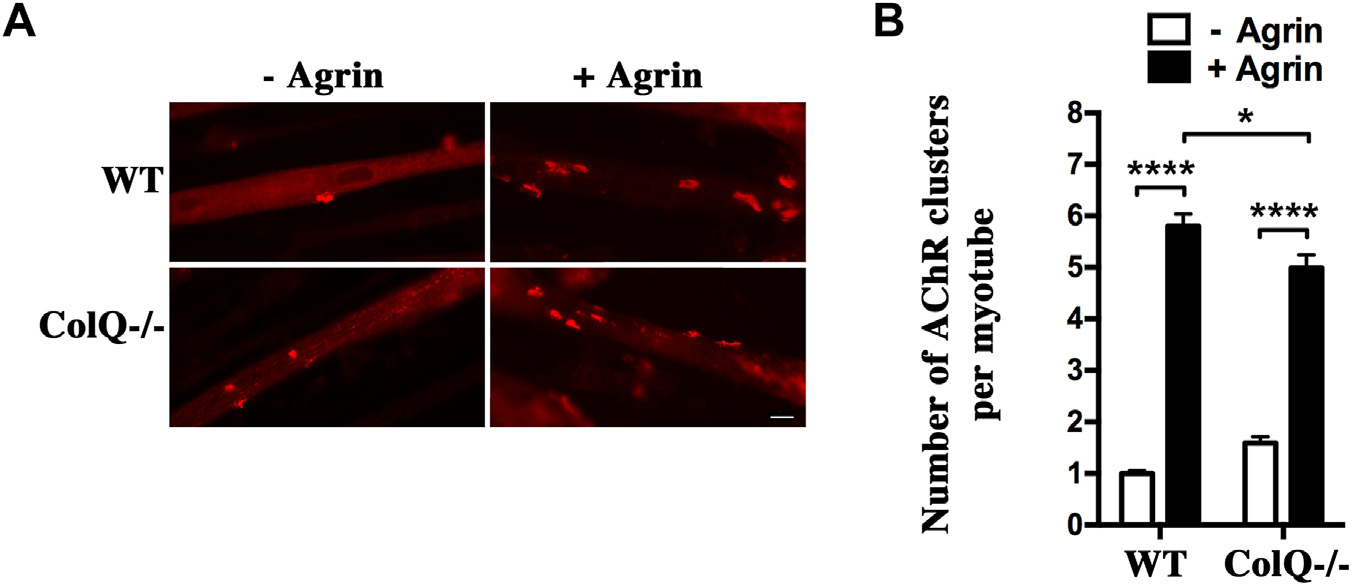
**ColQ regulates agrin-induced AChR clustering.***A*, WT and ColQ^−/−^ myotubes were treated or not with neural agrin (5 nM) for 16 h. AChR clusters were visualized by α-bungarotoxin staining. Scale bar represents 20 μm. *B*, the number of AChR clusters per myotube was quantified for each condition. At least, 60 myotubes from three independent experiments were analyzed for each condition. For quantification, a size threshold was applied such that only AChR clusters >5 μm^2^ were scored. The number of AChR clusters after agrin treatment was decreased by less than 15% in ColQ^−/−^ compared with WT myotubes. Results are expressed as the mean ± SEM. ∗*p* < 0.05; ∗∗∗∗*p* < 0.0001, using two-way ANOVA followed by Tukey's multiple comparison post hoc test. For interaction factor: F = 13.35, *p* = 0.0003; for −Agrin *versus* +Agrin: F = 455.2, *p* < 0.0001; for WT *versus* ColQ^−/−^: F = 0.32, *p* = 0.57 with a significant difference in the presence of agrin (*p* = 0.012) and no significant difference in the absence of agrin treatment. AChR, acetylcholine receptor; ColQ, collagen Q.

Then, we studied how ColQ modulation of MuSK phosphorylation affects AChR clustering. To this end, WT and ColQ^−/−^ myotubes were treated or not with neural agrin for 16 h, and the number of AChR clusters per myotube were quantified following α-bungarotoxin staining (Fig. 10*A*). Agrin stimulated AChR clustering both in WT and ColQ^−/−^ myotubes. The number of AChR clusters after agrin treatment appeared to be only moderately decreased by less than 15% in the absence of ColQ (Fig. 10*B*), which correlates with the weak reduction (less than 10%) of agrin-induced MuSK phosphorylation in ColQ^−/−^ compared with WT myotubes (Fig. 8, *A* and *B*). Overall, our results indicate that ColQ exerts a bivalent effect on agrin responsiveness, as we show that it reduces the level of agrin-induced MuSK phosphorylation but that this effect is largely compensated by an increase in cell surface levels of MuSK.

## Discussion

In addition to AChE anchoring, ColQ has regulatory functions at the NMJ that are mediated, at least in part, through MuSK (19). However, the molecular mechanisms, whereby ColQ interacts with the MuSK–LRP4 receptor complex and regulates its activity, still remained unclear. Using three different biochemical assays and SPR, we demonstrate that the only or main direct partner of ColQ within the MuSK–LRP4 complex at the NMJ is LRP4 and not MuSK. We also show, for the first time, that ColQ regulates agrin-induced MuSK phosphorylation.

A number of studies have reported that ColQ is able to bind to MuSK and that ColQ–MuSK interaction may participate in anchoring AChE–ColQ at the NMJ (9, 16, 17, 18, 49). This prompted us to compare ColQ–MuSK and ColQ–LRP4 interactions in our binding assays. Surprisingly, we could not detect any significant interaction between rat ColQ and rat ectoMuSK neither in our pull-down assays nor in our plate-binding assays, even when ectoMuSK was tested at much higher concentrations than ectoLRP4. Likewise, we did not detect any interaction between ColQ and purified MuSK in our SPR experiments. The rat- and quail-soluble ectodomains of MuSK have previously been used to demonstrate MuSK–LRP4 and MuSK–biglycan interactions, respectively, and thus appear to be correctly folded to interact with those proteins (37, 45). Therefore, it seems unlikely that the absence of interaction between ectoMuSK and ColQ results from any major misfolding of our rat MuSK ectodomain when expressed in solution, but it cannot be completely ruled out that soluble MuSK ectodomains have a reduced binding affinity for ColQ compared with full-length MuSK. It is noteworthy that another study failed to detect any AChE–ColQ binding to ectoMuSK, using a solid phase–immobilized MuSK ectodomain assay (45). In striking contrast, ectoMuSK and ColQ were coprecipitated in lysates from cotransfected heterologous cells, although at a much lower level than ectoLRP4 and ColQ, indicating that ectoMuSK is able to interact with ColQ in a cellular context as we observed for full-length MuSK and ColQ, in accordance with previous observations based on coimmunoprecipitation experiments (9, 17, 18). Unknown factors may be responsible for this weak ColQ–MuSK interaction in HEK and COS cells. Another possibility is that, although we did not detect any expression of LRP4 by Western immunoblot in HEK or COS cells, very small amounts of LRP4 are expressed and responsible for the coprecipitation of ColQ and MuSK in these cells, as LRP4 is also a direct binding partner of MuSK (36, 37).

Interestingly and consistent with this hypothesis, we found that LRP4 potentiates ColQ–MuSK interaction. This result suggests that LRP4, by forming a ternary complex with ColQ and MuSK, may stabilize a ColQ–MuSK interaction of very low affinity and quite unstable *in vitro*, and/or that LRP4 serves as an intermediate link between these two proteins enabling an indirect interaction. However, it should be noted, that some other previous studies reported the binding of AChE–ColQ to *in vitro*-immobilized ectoMuSK and MuSK, but the affinity constant was not determined and might be very low (16, 17, 49). The discrepancy with our results may arise from different experimental conditions. In particular, some of these studies used AChE–ColQ purified on heparin columns (16, 49), and not by sucrose gradients as in our case, that may contain some other heparin-binding proteins or factors associated with ColQ, which could indirectly stabilize ColQ–MuSK interactions. Alternatively, the apparently contradicting observations could be due to species differences, as these studies used human AChE–ColQ and MuSK, whereas our work was performed with murine proteins. Another possibility that cannot be excluded is that sequences from different MuSK isoforms were used. Indeed, MuSK isoforms that differ by the presence of alternatively spliced exons have been described, and these may present different affinities for binding partners (51, 52, 53).

Noteworthy, the contrasting results about ColQ–MuSK interaction can be put in perspectives with controversial data concerning the role of MuSK in AChE–ColQ anchoring at the NMJ. On the one hand, exogenous expression of MuSK restored the formation of AChE–ColQ clusters in MuSK-deficient myotubes (9). In addition, AChE–ColQ levels are reduced in WT mice receiving passively transferred anti-MuSK antibodies, which is interpreted as a consequence of the hindering of a ColQ–MuSK interaction (49). But on the other hand, AChE–ColQ accumulation is normal at NMJs in muscle biopsies of myasthenic patients with anti-MuSK autoantibodies that compromise ColQ–MuSK interaction, when tested *in vitro* (49, 54), suggesting that a possible ColQ binding to MuSK is not essential to the anchoring of AChE–ColQ at the NMJ, at least in humans. Moreover, in the biglycan null NMJs, where MuSK expression level is strongly decreased, AChE–ColQ levels remained unchanged (45). Thus, our results, showing a direct interaction between ColQ and LRP4, suggest that LRP4, which is localized at the synapse, may be key in AChE–ColQ anchoring and localization at the NMJ.

The C-terminal domain of ColQ, which does not interact with perlecan, has been shown to be essential for anchoring AChE–ColQ at the NMJ since most mutations in this domain lead to AChE deficiency in humans without abrogating the synthesis of AChE–ColQ hetero-oligomers (9, 10). However, the binding partners of this domain remain elusive. Here, we examined whether LRP4 might be the partner of the C-terminal domain of ColQ. We showed that a peptide corresponding to the 27 last C-terminal amino acids of ColQ is able to interact with LRP4 but, at the same time, we found that ColQ deleted of its 76 last C-terminal amino acids still interacts with the LRP4 ectodomain, although somewhat more weakly. Thus, at least in our *in vitro* conditions, the ColQ C-terminal domain is not essential for the ColQ–LRP4 interaction. It should be noted that this might not necessarily be the case *in vivo*, where ColQ might be oriented and inserted in the extracellular matrix (ECM) in a way where only its C terminus interacts with LRP4. Indeed, electron microscopy has revealed that AChE catalytic subunits associated to ColQ are positioned near the presynaptic membrane at the NMJ, suggesting that ColQ C terminus is localized close to the postsynaptic muscle membrane, whereas its collagenic domain crosses the synaptic cleft (55). Another possibility is that, as suggested by some previous studies, the binding partner of the ColQ C-terminal domain is MuSK (9, 16, 17, 18). According to our results, this interaction would be of very low affinity. Nevertheless, in line with our hypothesis of a ternary complex formed by LRP4 with ColQ and MuSK, it could be that LRP4 stabilizes a low-affinity interaction between the C-terminal domain of ColQ and MuSK that would, in cooperation with the ColQ–LRP4 interaction, be required to stably accumulate AChE–ColQ at the NMJ. This would correspond to a model where an optimal AChE–ColQ accumulation and anchoring at the NMJ would require both ColQ–LRP4 and ColQ–MuSK interactions. Alternatively, other unknown partners may be involved as different ColQ COOH-terminal mutants, causing congenital myasthenia with AChE deficiency, displayed a reduced binding to basement membrane extracts devoid of MuSK, suggesting that the anchoring function of the ColQ C terminus is in part independent of MuSK (17).

To better understand the underlying molecular mechanisms of ColQ signaling functions at the NMJ and since we observed that ColQ binds to the same N-terminal region of LRP4 as agrin, we decided to examine whether ColQ regulates the crucial agrin–LRP4–MuSK pathway. We observed that the total level of agrin-induced MuSK phosphorylation was slightly reduced in myotubes lacking ColQ. However, when taking into account that, in the absence of ColQ, cell surface MuSK levels were strongly reduced, in accordance with previous observations (19), it appears that the phosphorylation level of MuSK present at the cell surface of ColQ-deficient myotubes is in fact increased, compared with WT myotubes. Conversely, when ColQ was re-expressed in the ColQ^−/−^ myotubes, the phosphorylation level of MuSK was strongly decreased, indicating that the increase in agrin-induced MuSK phosphorylation observed in ColQ^−/−^ myotubes directly and specifically results from the absence of ColQ and not from some indirect effect. It should be noted that the levels of ColQ re-expressed after viral infection of ColQ^−/−^ myotubes may not exactly reflect the endogenous ColQ levels in WT myotubes, which may explain that in ColQ^−/−^ myotubes re-expressing ColQ, the level of agrin-induced MuSK phosphorylation was strongly decreased even before taking into account the increase in the cell surface level of MuSK.

Thus, ColQ has two opposing effects on MuSK activation. On the one hand, ColQ acts as a partial constitutive repressor of agrin-induced MuSK–LRP4 activation and, on the other hand, it upregulates and stabilizes MuSK at the muscle cell surface, consequently increasing the amount of potentially activated MuSK. These two opposing effects roughly balance each other, yielding a slight net decrease in total phosphorylated MuSK in response to agrin in the absence of ColQ. Consistent with this result and previous observations on AChR clustering and phosphorylation in ColQ^−/−^ myotubes in response to agrin (19), we observed a moderate reduction of agrin-induced AChR clustering in ColQ^−/−^ compared with WT myotubes. These data suggest that, in ColQ^−/−^ mice, the diminution of agrin-induced AChR clustering because of the decrease of membrane-bound MuSK would dominate over the enhancement of AChR clustering resulting from a higher MuSK activation. The decrease of AChR clustering, of MuSK plasma membrane levels and of MuSK activation, is also observed in various mutants of MuSK, LRP4, or Dok-7 that impair the agrin–MuSK–LRP4 pathway. This may explain why some patients with *COLQ* mutations share some common clinical features with those bearing mutations in *MUSK*, *LRP4*, and *DOK7* genes (14, 56, 57, 58).

Our results showing that ColQ strongly reduces agrin-induced MuSK phosphorylation level in HEK cells cotransfected with only MuSK and LRP4 suggest that ColQ acts directly on agrin, MuSK, and/or LRP4 and that no other muscle-specific factor is required. However, we can exclude that ColQ acts through an interaction with agrin since we have shown that ColQ does not interact with the recombinant neural agrin that was used in our binding and phosphorylation assays. In the absence of any stable direct interaction between ColQ and MuSK, our results rather suggest that ColQ partially reduces agrin-induced MuSK–LRP4 activation *via* its attachment to LRP4. One possibility is that ColQ–LRP4 interaction decreases agrin binding to LRP4. This is supported by our data indicating that ColQ binds to the N-terminal region of LRP4, which is known to contain the agrin-binding sites (50), and by our results revealing that agrin and ColQ compete for their binding to LRP4. Whether ColQ and agrin compete to some extent for partially overlapping or nearby binding sites through steric hindrance or whether ColQ binds to allosteric sites to modulate the affinity of LRP4 for agrin remains to be investigated. Alternatively, ColQ may modify the agrin-induced LRP4 conformational changes required for MuSK activation. Another possibility is that ColQ reduces the agrin-induced MuSK–LRP4 coupling, as suggested by results from Otsuka *et al*. (18) showing that ColQ reduced MuSK–LRP4 interaction using an *in vitro* plate-binding assay. According to the authors, ColQ and LRP4 may compete for the same binding regions on MuSK. Instead, if considering that ColQ and MuSK do not interact directly, it could be that ColQ and MuSK compete for the same binding region on LRP4. This is suggested by our results showing that the third β-propeller domain, which is important for LRP4 association with MuSK (50), is also involved in ColQ–LRP4 interaction, although to a lesser extent than the N-terminal region of LRP4.

Thus, the precise mechanisms whereby ColQ reduces MuSK–LRP4 activation remain to be elucidated and will require to identify more precisely the interaction domains of ColQ with LRP4 in future studies. It would also be of interest to evaluate whether ColQ or LRP4 identified mutations or autoantibodies, responsible for congenital myasthenia or myasthenia gravis, disrupt or diminish the interaction between LRP4 and ColQ. It also remains unexplored whether ColQ modulates the response of the MuSK–LRP4 complex to other ligands, such as Wnts or biglycan. Finally, it should be considered that, in ColQ-mutated congenital myasthenic syndromes, the absence of ColQ may also affect signaling pathways at the NMJ by indirect mechanisms, in addition to its direct modulation of the MuSK–LRP4 complex. Indeed, one of the hallmark of ColQ-deficient NMJs is the perturbation of the ECM organization (13). In a previous study, we also found that ColQ deficiency modified the mRNA levels of major ECM components in muscle cells (59).

In conclusion, our results reveal that LRP4 is a receptor for ColQ at the NMJ. Our data provide new insights into the molecular mechanisms underlying ColQ signaling and AChE-anchoring functions at the NMJ. They also have implications for our understanding of myasthenic syndromes, which may be linked in some cases to ColQ or LRP4 mutations or autoantibodies disrupting or weakening ColQ–LRP4 interaction.

## Experimental procedures

### Antibodies and reagents

Mouse monoclonal anti-Flag M2, anti-GFP, anti-α tubulin antibodies, and IgG1 isotype control from murine myeloma were purchased from Sigma–Aldrich. Mouse monoclonal anti-Myc Tag (9B11), mouse monoclonal anti-His Tag (27E8), and rabbit polyclonal anti-Flag antibodies were purchased from Ozyme. Rabbit polyclonal anti-HA and rabbit monoclonal anti-placental AP antibodies were from Abcam. Mouse monoclonal anti-LRP4 antibodies were purchased from Neuromab, whereas rabbit polyclonal anti-LRP4 antibodies were from Sigma–Aldrich. Rabbit polyclonal anti-MuSK antibodies were from Millipore or Abcam. Monoclonal antiphosphotyrosine (clone 4G10) antibodies were from Millipore. Mouse monoclonal anti-TfR antibody and Alexa Fluor 594–conjugated α-bungarotoxin were from Invitrogen. Recombinant rat neural agrin, mouse ectoLRP4 His-tag, and mouse ectoMuSK Fc Chimera were from R&D Systems. Horseradish peroxidase–conjugated goat antimouse IgG light chains were from Jackson Immunoresearch. Horseradish peroxidase–conjugated goat anti-rabbit IgG was from GE Healthcare. Dynabeads Protein G and Dynabeads His-tag were purchased from Invitrogen.

### Constructs

ColQ-Flag plasmid constructs were generated by the introduction of a sequence encoding the Flag epitope after the triplet coding for amino acid 26 in the rat ColQ1a cDNA (previously described (60)) inserted into a pcDNA3 plasmid. ColQΔCt-Flag was generated using PCR mutagenesis by deleting the C-terminal sequence from amino acid 375 to 451 (end). ColQ-GFP constructs were previously described (9) and were obtained by the introduction of a GFP tag sequence after the triplet coding for amino acid 20 in the rat ColQ1a cDNA. ColQ-Myc corresponds to mouse ColQ1a cDNA with a Myc tag at the COOH terminus. A Flag-tagged peptide corresponding to the last 27 amino acids of the C-terminal domain (sequence of exon 17 plus four amino acids of exon 16) of rat ColQ1a (Flag-ColQ Cter [aa 425–451]) was produced by Genecust (Luxembourg). Human AdipoQ-Flag was from Origene. Rat AChE_T_ sequence was inserted into a pEF-BOS plasmid. LRP4-Myc was generated by adding a C-terminal Myc tag to the rat LRP4 cDNA sequence by PCR mutagenesis using Pfu UltraII DNA polymerase (Agilent). EctoLRP4-Myc corresponds to the extracellular domain of rat LRP4 fused to one Myc tag and followed by a 6xHis tag at the COOH terminus and was a gift from Lin Mei (37). LRP6-Myc was a gift from Bruno Canque and corresponds to human LRP6 cDNA with one Myc tag placed at the COOH terminus, and ectoLRP6-Myc was generated from it by deleting the intracellular and transmembrane domains by PCR mutagenesis. Rat MuSK and MuSK-HA (HA tag at the COOH terminus) cDNAs were previously described (9). We generated a rat MuSK-Myc construct with an unique Myc tag followed by a 6xHis-tag at the COOH terminus. EctoMuSK-Myc was generated by PCR deletion of MuSK-Myc intracellular and transmembrane domains. Human placental AP, mouse ectoLRP4-AP, and rat ectoMuSK-AP sequences were all in pcDNA3 and were gifts from Steven Burden (50). The mouse ectoLRP4-AP deletion mutants were generated by PCR mutagenesis. EctoLRP4Δ1-AP was obtained by deleting the sequence located between amino acids 25 and 736, including the eight LDLa repeats, the two first EGF-like domains followed by the first β-propeller domain, whereas ectoLRP4Δ2-AP was deleted from the amino acids 737 to 1049 containing the second β-propeller domain. EctoLRP4Δ3-AP and ectoLRP4Δ4-AP lacked amino acids from 1048 to 1353 (including the third β-propeller domain) and from 1353 to 1693 (including the fourth β-propeller domain), respectively. EctoLRP4Δ234-AP corresponds to the deletion from amino acids 737 to 1693 (Fig. 7*A*). Unfortunately, ectoLRP4Δ2-AP and ectoLRP4Δ4-AP were not efficiently secreted by transfected cells and could not be tested. Plasmids were purified using NucleoSpin Plasmid kit (Macherey-Nagel). Oligonucleotides were synthesized by Eurofins. All constructs were verified by DNA sequencing (Eurofins). rAAV2 vectors, containing the cDNA sequence coding for human ColQ1a with a Myc tag inserted after the triplet coding for amino acid 24 and the cDNA coding for EGFP separated by the linker IRES (rAAV2-ColQ-Myc) or containing only the sequence coding for EGFP (rAAV2-EGFP), were produced by VectorBuilder.

### HEK 293T and COS-7 cell culture and transfection

HEK 293T and COS-7 cells, which do not express detectable levels of LRP4 or MuSK (data not shown), were cultured in Dulbecco's modified Eagle's medium (DMEM) (Fisher Scientific) supplemented with 10% fetal calf serum (FCS), 2 mM l-glutamine, and penicillin/streptomycin (5000 U) at 37 °C in 5% CO_2_ and passaged 24 h before transfection at a density to reach about 70% confluence next day in 10 cm dishes. They were transfected with Fugene HD (Promega) according to the manufacturer's instructions with a DNA/Fugene ratio of 1 μg:3 μl. For cotransfections, 2 μg of each construct were used unless otherwise stated. In the case of ColQ transfection, cell culture medium was supplemented with 50 μg/ml of ascorbic acid (Sigma) to stimulate collagen synthesis. Cells were lysed 48 h after transfection.

### Muscle cell culture and transduction

The two muscle cell lines, WT and ColQ-deficient (ColQ^−/−^), were generated as previously described (9). Myoblasts were maintained in DMEM supplemented with 10% fetal bovine serum, 20% horse serum, 2 mM glutamine, 2% penicillin/streptomycin (5000 U), and 20 U/ml of γ-interferon (Roche Diagnostics) at 33 °C in 8% CO_2_. Cells were then cultured on plates coated with collagen type I and differentiated into myotubes at 37 °C in 5% CO_2_ in DMEM supplemented with 5% horse serum and without γ-interferon (differentiation medium) for 7 days unless otherwise stated. ColQ-Myc was expressed in ColQ^−/−^ myotubes by transduction with the rAAV2-ColQ-Myc recombinant AAV. As a control, ColQ^−/−^ myotubes were infected with the rAAV2-EGFP virus expressing only EGFP. Briefly, after 1 day in differentiation medium, cells were washed to remove serum, and the virus (20,000 multiplicity of infection) was added to cells twice at 5 h intervals in a minimal volume of DMEM. Two hours later, the culture medium was supplemented with differentiation medium, and myotubes were harvested 4 days post-transduction.

### Recombinant protein production and purification

For the production of AP and LRP4, LRP6, or MuSK extracellular domains, DMEM 10% FCS cell culture medium was replaced 24 h after transfection with Opti-MEM reduced serum medium (Fisher Scientific) supplemented with 0.01% of FCS. Cells transfected with ectoLRP4 constructs were grown at 32 °C to increase the secretion of the corresponding protein, which turned out to be very weak at 37 °C. The next day, media were collected, concentrated 30-fold using Amicon Ultra-30 centrifugation filters (Millipore), and stored at 4 °C in the presence of protease inhibitors and 0.05% sodium azide (for AP fusion proteins, sodium azide was not added). For the collection of cell lysates, 48 h after transfection, cells were washed with PBS, scraped in lysis buffer (50 mM Tris, 150 mM NaCl, 3 mM EDTA, 20 mM NaF, 1% [v/v] Triton X-100, pH 7.4) supplemented with a cocktail of protease inhibitors (Roche Diagnostics) and kept shaking on ice for further 30 min. The extracts were centrifuged at 20,000*g* at 4 °C for 15 min, and the supernatants were frozen at −20 °C. Protein concentrations were determined using the bicinchoninic acid reagent kit assay (Pierce Biotechnology). For some experiments, the 6xHis-tagged ectoLRP4-AP, ectoLRP4-Myc, or ectoMuSK-Myc were purified from CM prepared with an EDTA-free cocktail of protease inhibitors using the magnetic Dynabeads His-tag according to the manufacturer's instructions.

### Production and purification of AChE forms by sucrose density gradients

HEK 293T cells cotransfected with 5 μg ColQ-Flag and 5 μg AChE were lysed 48 h later in 25 mM Tris–HCl (pH 7.4), 0.8 M NaCl, 1% CHAPS with the following protease inhibitors: 10 mM EDTA, 40 μg/ml leupeptin, 10 μg/ml pepstatin, and 2 mM benzamidine. After homogenization in a Teflon-glass Dounce homogenizer for 3 min, the extracts were held on ice for 1 h followed by a centrifugation at 20,000*g* for 15 min. The different AChE forms were separated in 5 to 20% (w/v) sucrose gradients containing 50 mM Tris–HCl, pH 7, 0.8 M NaCl, 10 mM EDTA, and 0.2% Brij-97 (polyoxyethylene 10 oleoyl ether; Sigma–Aldrich) as described (61). The gradients were ultracentrifuged at 38,000 rpm at 7 °C for 18.5 h, using a SW41 rotor (Beckman Instruments). Each gradient was collected in 48 fractions and assayed for AChE activity by the Ellman method (62). Fractions were calibrated with the internal sedimentation markers: AP (6.1S) and β-galactosidase (16S). Sedimentation marker profiles were used to establish a linear relation between fraction number and Svedberg units. HEK 293T cells produced trimeric ColQ forms (corresponding to AChE–ColQ forms A12, A8, and A4 with respectively 3, 2, and 1 AChE tetramers associated to ColQ; not shown). Fractions corresponding to A12 forms and G1 globular monomeric ColQ-free AChE forms were collected and diluted with Tris–HCl buffer to obtain preparations with a final NaCl concentration of 0.2 M for binding experiments.

### Coimmunoprecipitation

Cells were harvested in a lysis buffer (50 mM Tris, 150 mM NaCl, 3 mM EDTA, 20 mM NaF, 1% [v/v] Triton X-100, pH 7.4) supplemented with a cocktail of protease inhibitors (Roche Diagnostics) for 30 min at 4 °C. Insoluble material was removed by centrifugation at 20,000*g* at 4 °C for 15 min. Immunoprecipitation was performed on 1 to 1.5 mg of total protein content in a volume of 500 μl with 4 μg of indicated antibodies after a preclear step. Immunocomplexes were precipitated with 50 μl of protein G magnetic beads (Dynabeads) according to the manufacturer’s instructions for 3 h at 4 °C. Immunoprecipitates were then washed in lysis buffer with a final wash in 50 mM Tris buffer (pH 7.4) and resuspended in 1× Laemmli sample buffer at 70 °C before analysis by Western immunoblot.

### Pull-down assay

ColQ-Flag from transfected HEK 293T cell lysates was immobilized for 2 h on protein G magnetic beads precoated with anti-Flag antibodies. Control beads correspond to beads precoated with anti-Flag that were incubated with the same protein concentrations of HEK 293T cell lysates from nontransfected cells. The beads were then incubated with purified ectoLRP4-AP, ectoLRP4-Myc, or ectoMuSK-Myc or with CM containing ectoLRP4-AP, ectoLRP4-Myc, ectoLRP6-Myc, or ectoMuSK-Myc at 4 °C overnight. Beads were then washed three times with lysis buffer and one time with 50 mM Tris buffer. Bound proteins were eluated with 1× Laemmli sample buffer at 70 °C and analyzed by Western immunoblot. In other experiments, same amounts of ectoLRP4-Myc, ectoLRP6-Myc, or ectoMuSK-Myc were immobilized to protein G magnetic beads precoated with anti-Myc antibodies. Control beads correspond to beads precoated with anti-Myc that were incubated with CM of untransfected HEK 293T cells. The beads were then incubated with 0.25 Ellman units of purified AChE–ColQ (A12 forms) or ColQ-free AChE (G1 forms) at 4 °C overnight. After extensive washing with PBS, bound AChE activity was quantified by the Ellman method as described (61).

### Plate-binding assay

ColQ-Flag or AdipoQ-Flag from transfected HEK 293T cell lysates was immobilized overnight at 4 °C on anti-Flag precoated wells of a 96-well microtiter assay plate (Flag Tag Antibody Plate from GenScript). It was checked that AdipoQ-Flag was bound at least as much as ColQ-Flag. Control wells were incubated with same protein concentrations of untransfected cell lysates. In some experiments, wells were coated with sucrose gradient–purified AChE–ColQ-Flag (A12 forms) or with the same sucrose gradient fractions obtained from untransfected cells as a control. After washing, wells were incubated with 200 μl of the appropriate dilutions of AP or AP fusion proteins in Tris-buffered saline (TBS)–0.05% Tween, 1 mM MgCl_2_ overnight at 4 °C to ensure that the binding reactions have reached the equilibrium state as determined by equilibrium measurements (not shown). Within an assay, a constant level of AP activity, corresponding to fusion protein concentrations ranging from 10 to 100 nM according to the type of experiment, was added to the wells. For competition with agrin, ColQ-coated wells were incubated with 25 nM of ectoLRP4-AP in the presence of 500 nM agrin or bovine serum albumin (BSA) as a control. Plates were washed four times with TBS–0.05% Tween, 1 mM MgCl_2_, and the amount of AP bound to the wells was measured using *p*-nitrophenyl phosphate as a substrate (Sigma–Aldrich). The yellow reaction product was read at 405 nm in an Enspire (PerkinElmer) microtiter plate spectrophotometer.

### SPR

The SPR biosensor experiments were performed on a Biacore 3000 instrument (Cytiva) in the molecular interactions facility of the Institute of Biology Paris Seine (Sorbonne University). First, mouse monoclonal anti-Flag M2 (Sigma–Aldrich), diluted at 50 μg/ml in 10 mM sodium acetate (pH 5.5), was covalently coupled (about 14,000 RUs) *via* its primary amino groups to the carboxymethyl dextran matrix of a CM5 sensor chip (Cytiva). Free activated sites of the matrix were saturated by injection of ethanolamine hydrochloride (1 M) pH 8.5. HBS–EP running buffer (10 mM Hepes [pH 7.4], 150 mM NaCl, 3 mM EDTA, 0.005% P20 surfactant) was used as running buffer and to dilute all the injected molecules. ColQ-Flag from transfected HEK 293T cell lysates was diluted in the running buffer and then injected into dedicated channels at a flow rate of 5 μl/min for 30 min and dissociated for at least 10 min to stabilize ColQ binding and the baseline. About 3900 RUs of bound ColQ (the ligand) were obtained. Reference sensor surfaces were obtained using the same procedure by injecting preparations from untransfected cells at the same protein concentration as ColQ-Flag preparations (a limited nonspecific binding [320 RUs] compared with ColQ was detected). The relative responses indicated in RU were calculated using the response values of report points corresponding to 30 s of dissociation. To study the interaction of ectoLRP4 (the analyte) with ColQ, SCK experiments were performed as regeneration steps would alter the noncovalent ColQ capture on the sensor surface. Increasing concentrations of purified ectoLRP4-His were sequentially passed over ColQ-bound or reference surfaces at a flow rate of 5 μl/min with an association phase of 3 min followed by a dissociation phase of 5 min for each concentration. Binding curves were corrected by substraction of the SPR signals obtained for the reference surface from those obtained with the ColQ-bound surface. A second reference was performed by repeating the same sequence of injections with running buffer instead of ectoLRP4, and the corresponding drift signals were subtracted from the sensorgrams to obtain the accurate binding profiles prior to the kinetic analysis. SCK data were processed by fitting the binding profiles to a 1:1 binding model with drifting baseline using the BIAevaluation software, version 4.1 (Biacore AB). From the fitted data, the association (*k*_on_), dissociation (*k*_off_), and equilibrium dissociation (*K*_*d*_) constants were calculated.

### Electrophoresis and Western immunoblot

Proteins were separated on NuPAGE Novex 7% Tris-acetate or 4-20% Tris-glycine polyacrylamide gels (Invitrogen, Fisher Scientific) under reducing conditions before overnight electrophoretic transfer onto nitrocellulose membranes (Bio-Rad). The blots were blocked for 1 h in TBS–0.05% Tween-20 with 5% nonfat dried milk. All subsequent steps were performed with this blocking buffer at room temperature, except for incubation with antiphosphotyrosine antibodies performed in TBS–0.05% Tween-20 with 3% BSA. The blots were then incubated with the primary antibodies: mouse anti-Flag (1:1500 dilution), anti-Myc (1:1000 dilution), anti-α tubulin (1:6000 dilution), anti-TfR (1:500 dilution), antiphosphotyrosine (1:1000 dilution), rabbit anti-HA (1:2000 dilution), anti-MuSK (1:1000 dilution), or anti-LRP4 (1:1000 dilution) for 1 to 2 h. Blots were then incubated with peroxidase-conjugated anti-mouse IgG light chains (1:15,000 dilution) or anti-rabbit IgG (1:20,000 dilution) antibodies before exposure to the chemiluminescent substrate ECL prime (GE Healthcare). Relative signal intensity of the different bands was measured by densitometry using ImageJ software (NIH).

### MuSK phosphorylation assay

Cells were incubated in serum-free medium for 3 h before they were treated or not with 10 nM of recombinant rat neural *Sf*21–derived C-terminal agrin (R&D Systems) for 1 h. Cells were lysed in radioimmunoprecipitation assay buffer supplemented with a cocktail of protease inhibitors, 20 mM NaF, and 5 mM orthovanadate. After centrifugation, soluble material was precleared with protein G magnetic beads and incubated with anti-MuSK antibody or anti-HA antibody for MuSK-HA. Immunocomplexes were precipitated with 50 μl of protein G magnetic beads for 3 h at 4 °C. Immunoprecipitates were then washed in lysis buffer, eluated in 1× Laemmli sample buffer at 70 °C, electrophoresed on 7% Tris-acetate gels, transferred to nitrocellulose, and probed with anti-phosphotyrosine (clone 4G10) antibodies. Blots were then stripped and reprobed with anti-MuSK or anti-HA antibodies.

### Biotinylation and separation of cell surface proteins

HEK 293T cells or myotubes before or after agrin treatment were washed with PBS containing 0.1 mM CaCl_2_ and 1 mM MgCl_2_ (PBS–Ca–Mg) and incubated with 0.5 mg/ml EZ-Link Sulfo-NHS-SS-Biotin (Thermo Fisher Scientific) in PBS–Ca–Mg at 4 °C for 30 min. Cells were then rinsed twice with ice-cold PBS–Ca–Mg and once with PBS before protein extraction in radioimmunoprecipitation assay lysis buffer. Biotinylated proteins were recovered using streptavidin–agarose beads (Thermo Fisher Scientific). After four washes, bound proteins were eluated by heating beads at 75 °C in 1× Laemmli sample buffer. Cell surface MuSK and LRP4 present in the precipitates were then analyzed by Western immunoblot. The membrane TfR was used as a loading control to normalize the results.

### AChR clustering assays

After 5 days of differentiation, WT and ColQ-deficient myotubes were stimulated or not with 5 nM of recombinant rat neural agrin (R&D Systems) for 16 h. Muscle cells were fixed in 4% paraformaldehyde and incubated with Alexa Fluor 594–conjugated α-bungarotoxin (1:1000 dilution) for 1 h. Images were collected using a microscope (Olympus BX61) equipped with a Fast 1394 Digital CCD FireWire camera (model Retiga 2000R; Qimaging) and a 40× oil immersion objective (numerical aperture: 1.0; Olympus). For quantification, a size threshold was applied such that only AChR clusters >5 μm^2^ were scored.

### Statistical analysis

Data were analyzed in GraphPad Prism (GraphPad Software, Inc). For the pull-down and plate-binding assays, the means of three or more independent groups split on two variables or factors were compared using two-way ANOVA followed by Tukey's multiple comparison post hoc test. For Western immunoblots, quantifications data were normalized to the control group as to obtain fold changes and were analyzed using the one-sample *t* test when data of one group were compared with the control set as one or 100% with no variance. Data are presented as mean ± SEM and were considered significant when *p* < 0.05. *p* Values are displayed graphically as follows: ∗ for *p* < 0.05, ∗∗ for *p* < 0.01, ∗∗∗ for *p* < 0.001, and ∗∗∗∗ for *p* < 0.0001.

## Data availability

All data are contained within the article.

## Conflict of interest

The authors declare that they have no conflicts of interest with the contents of this article.
